# A Huaxian Formula Nanoformulation Confers Protection Against Acute Radiation‐Induced Lung Injury by Targeting Ferroptosis

**DOI:** 10.1002/advs.77472

**Published:** 2026-08-29

**Authors:** Li Quan, Junyang Chen, Xingmin Chen, Zengyi Fang, Yuanzhen Mi, Pingjin Zou, Shunlian Fu, Huakang Li, Ke Xu, Bing Lin, Jinyi Lang, Meihua Chen

**Affiliations:** ^1^ Department of Radiation Oncology Precision Radiation in Oncology Key Laboratory of Sichuan Province Sichuan Clinical Research Center for Cancer Sichuan Cancer Center Sichuan Cancer Hospital & Institute Affiliated Cancer Hospital of University of Electronic Science and Technology of China Chengdu China; ^2^ School of Clinical Medicine Chengdu University of Traditional Chinese Medicine Chengdu China; ^3^ School of Medicine University of Electronic Science and Technology of China Chengdu China

**Keywords:** acute radiation‐induced lung injury, ferroptosis, Huaxian formula, nanoformulation, oxidative stress

## Abstract

Acute radiation‐induced lung injury (RILI) remains a major dose‐limiting complication of thoracic radiotherapy with limited therapeutic options. Here, we develop an inhalable nanoformulation of the traditional Chinese medicine Huaxian Formula (NHXF) with favorable physicochemical stability and enhanced pulmonary delivery properties. In vitro and in vivo studies demonstrate that NHXF attenuates radiation‐induced alveolar epithelial injury, accompanied by reductions in oxidative stress and apoptosis. NHXF also suppresses secondary inflammatory responses, as evidenced by decreased pro‐inflammatory cytokine production and neutrophil infiltration, which may contribute to its protective effects against acute RILI. Importantly, NHXF displays a favorable safety profile throughout treatment. Transcriptomic analysis highlights ferroptosis‐related pathways associated with NHXF‐mediated protection. Our findings indicate that NHXF treatment is associated with the attenuation of ferroptosis‐related alterations in iron homeostasis and lipid peroxidation. Pharmacological activation of ferroptosis significantly diminishes the protective effects of NHXF against radiation‐induced injury, further supporting a functional contribution of ferroptosis modulation to NHXF‐mediated protection, while acknowledging that additional direct mechanistic evidence is warranted to establish causal dependency. Collectively, these findings support NHXF as a potential inhalable nanotherapeutic for acute RILI and highlight pulmonary delivery of nanoformulated traditional Chinese medicine combined with ferroptosis modulation as a promising strategy for RILI management.

## Graphical abstract

An inhalable, nanoengineered herbal therapy achieves protection against acute radiation‐induced lung injury via self‐assembled nanomicelle‐mediated pulmonary targeting and a previously unrecognized dual mechanism that attenuates oxidative stress while inhibiting ferroptosis in alveolar epithelial cells.

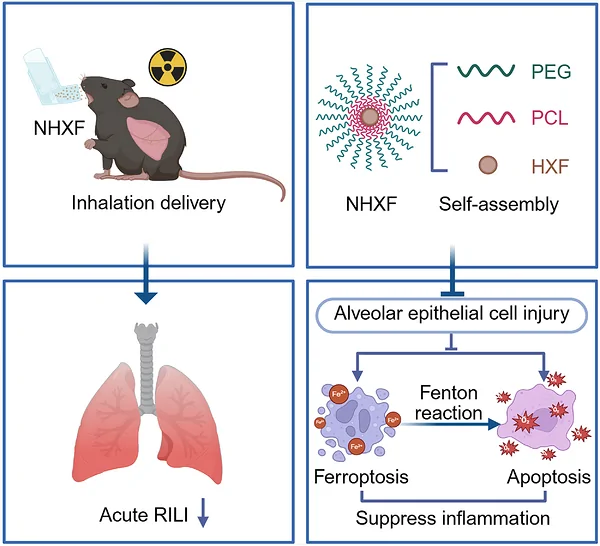

## 1 Introduction

Radiotherapy remains a cornerstone in the treatment of thoracic malignancies, including lung, esophageal, and breast cancers [1, 2]. However, its therapeutic efficacy is frequently constrained by radiation‐induced lung injury (RILI), a potentially severe dose‐limiting toxicity that manifests as pneumonitis and pulmonary fibrosis, ultimately compromising patient outcomes [3]. RILI follows a distinct pathological progression, beginning with an acute inflammatory phase characterized by radiation pneumonitis (RP) and subsequently advancing to irreversible radiation‐induced pulmonary fibrosis (RIPF). The fibrotic stage is marked by progressive extracellular matrix deposition and tissue remodeling, resulting in irreversible loss of pulmonary function [4]. Since the severity of acute lung injury critically influences subsequent fibrotic remodeling, early therapeutic intervention during the acute phase is essential for interrupting disease progression [5]. Despite the clinical importance of radioprotection, no pharmacological intervention has yet demonstrated consistently validated efficacy and safety [6]. Corticosteroids remain the first‐line treatment for symptomatic RILI; however, their benefits are largely transient and fail to alter the underlying pathogenic processes. Moreover, prolonged administration is frequently associated with substantial adverse effects, underscoring the urgent need for alternative therapeutic strategies [3, 7].

Traditional Chinese Medicine (TCM), characterized by its holistic therapeutic philosophy and multi‐component pharmacology, represents a valuable source for therapeutic discovery [8]. The Huaxian Formula (HXF), a classical multi‐herbal prescription refined through extensive clinical practice, has demonstrated promising efficacy in alleviating RILI and is believed to exert its effects through coordinated regulation of multiple pathological pathways [9, 10]. However, its broader application is limited by unfavorable physicochemical properties, including poor aqueous solubility, low bioavailability, and inadequate pulmonary accumulation following systemic administration [11, 12, 13]. These limitations necessitate relatively high dosing, contribute to variable therapeutic outcomes, and impede its clinical translation. To overcome these challenges, nanomedicine strategies based on biodegradable copolymers such as poly(ethylene glycol)‐block‐poly(ε‐caprolactone) (PEG‐PCL) have been widely explored to enhance drug solubility, stability, and pharmacokinetic performance [14]. Nevertheless, current nanomedicine applications in TCM remain largely focused on single active constituents. Such reductionist delivery strategies fail to preserve the intrinsic multi‐component synergy of TCM formulations and are therefore insufficient to address the complex pathophysiology of RILI [15, 16].

Among the molecular mechanisms implicated in RILI, oxidative stress and ferroptosis have emerged as closely interconnected pathological processes. Ionizing radiation triggers excessive production of reactive oxygen species (ROS), overwhelming endogenous antioxidant defenses and causing extensive damage to lipids, proteins, and nucleic acids [17]. Simultaneously, radiation disrupts cellular iron homeostasis, leading to the accumulation of labile iron that further amplifies oxidative stress through Fenton reactions [18, 19]. The combined elevation of ROS and intracellular iron promotes excessive lipid peroxidation, ultimately triggering ferroptosis, an iron‐dependent form of regulated cell death characterized by the accumulation of lipid peroxides and oxidative membrane damage. Ferroptosis is governed by a complex network of antioxidant defense systems and lipid metabolic pathways. Among these, the GPX4‐centered axis represents a major protective mechanism by catalyzing the reduction of lipid hydroperoxides to lipid alcohols and thereby limiting lipid peroxidation propagation [20, 21]. Conversely, ACSL4 enhances ferroptotic vulnerability by promoting the incorporation of polyunsaturated fatty acids into membrane phospholipids, thereby enriching membranes with peroxidation‐prone lipids [22, 23]. Accordingly, therapeutic strategies targeting ferroptosis have attracted increasing attention as a mechanistically rational approach for mitigating RILI [24, 25]. However, effective ferroptosis‐targeting therapeutics with robust pulmonary delivery capability remain largely unavailable.

Here, we propose an integrated therapeutic strategy that combines the multi‐component pharmacological characteristics of TCM with nanomedicine‐based pulmonary delivery and mechanistic targeting of ferroptosis. To this end, HXF is incorporated into a PEG‐PCL copolymer‐based nanocarrier to generate a nanoformulated HXF (NHXF), with the aim of improving bioavailability and enabling targeted pulmonary delivery via nebulized inhalation. We further hypothesize that the therapeutic benefits of NHXF extend beyond enhanced drug delivery and involve coordinated regulation of oxidative stress and ferroptosis‐related pathways. Accordingly, this study systematically evaluates the radioprotective efficacy of NHXF and elucidates its underlying molecular mechanisms, aiming to establish a mechanistically validated nanotherapeutic candidate for the prevention and treatment of acute RILI.

## 2 Results and Discussion

### 2.1 Preparation and Characterization of PEG‐PCL‐HXF (NHXF)

**FIGURE 1:**
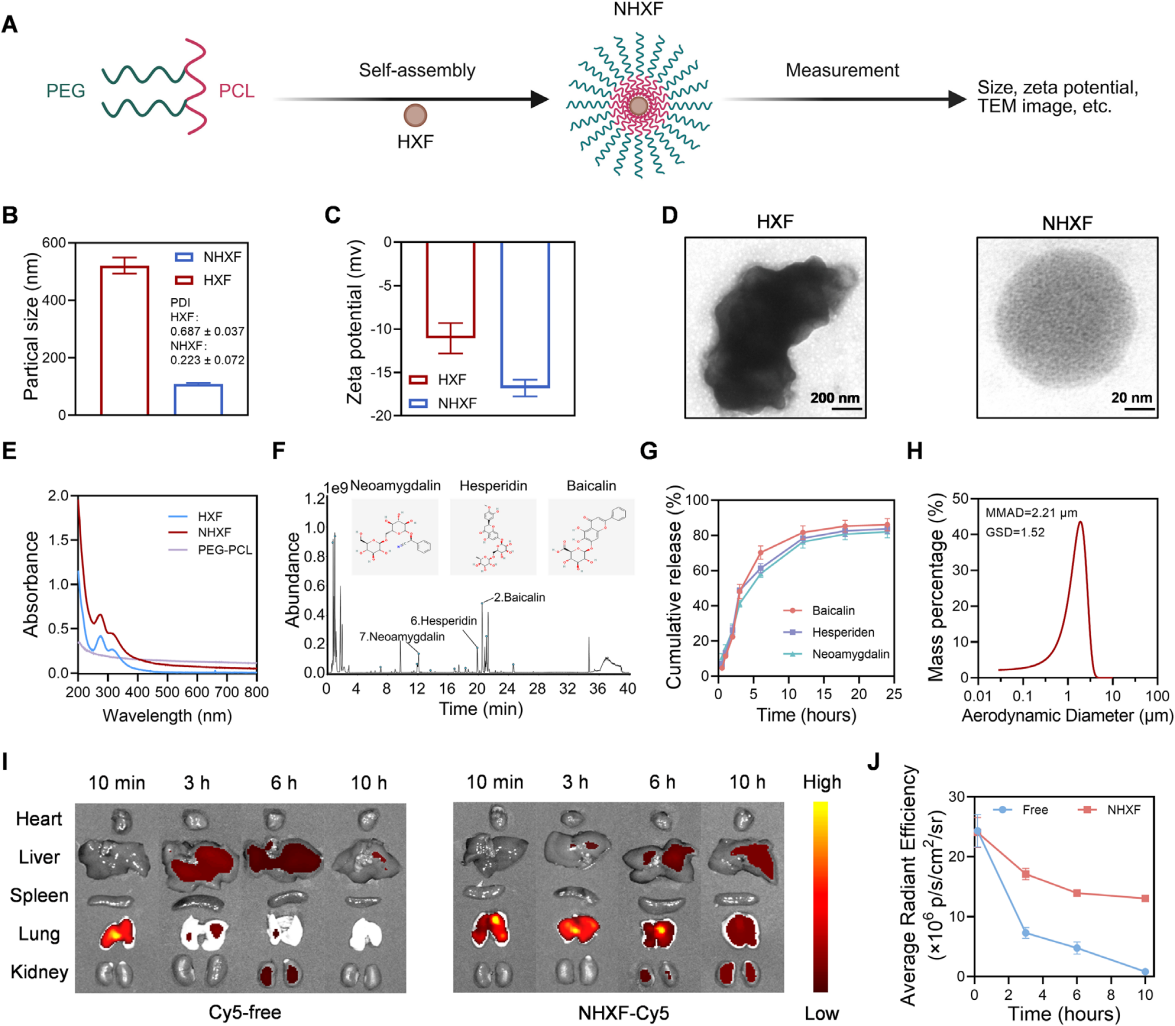
Preparation and physicochemical characterization of NHXF. (A) Schematic illustration of the self‐assembly process of NHXF from PEG‐PCL copolymer and HXF components. (B) Hydrodynamic size distribution and (C) zeta potential of NHXF and the parent HXF decoction, as determined by DLS. Data are presented as mean ± SD (*n* = 3 independent preparations). (D) Representative TEM images of the HXF decoction and NHXF nanoparticles. Scale bars: 200 nm (HXF) and 20 nm (NHXF). (E) UV‐vis absorption spectra of HXF and NHXF. (F) Base peak chromatogram (BPC) of NHXF obtained by HPLC‐MS, highlighting representative encapsulated bioactive constituents, including neoamygdalin, hesperidin, and baicalin. (G) Cumulative release profiles of neoamygdalin, hesperidin, and baicalin in simulated alveolar fluid (37°C, pH 7.4). Data are presented as mean ± SD (*n* = 3 independent preparations). (H) Aerodynamic particle size distribution of nebulized NHXF determined by cascade impaction. (I) Representative ex vivo fluorescence images showing the biodistribution of free Cy5 and Cy5‐labeled NHXF in major organs at the indicated time points after nebulized inhalation. (J) Quantification of lung fluorescence intensity based on ex vivo region‐of‐interest (ROI) analysis. Data are presented as mean ± SD (*n* = 3).

Traditional Chinese Medicine has shown considerable therapeutic potential in the management of RILI [26]. However, the clinical application of conventional herbal decoctions such as HXF remains limited by inherent drawbacks, including poor aqueous solubility and low bioavailability [16]. To address these challenges, we developed a nanoformulation of HXF using the biocompatible amphiphilic block copolymer PEG‐PCL (Figure 1A). The hydrophobic PCL segment encapsulates the hydrophobic constituents of HXF through self‐assembly and noncovalent interactions, while the hydrophilic PEG chains form a stabilizing outer corona, thereby enhancing aqueous dispersibility and colloidal stability. To verify successful nanoformulation, particle size and distribution were characterized. Dynamic light scattering (DLS) revealed that NHXF nanoparticles exhibited a hydrodynamic diameter of 108.83 ± 3.85 nm with a narrow size distribution (PDI = 0.223 ± 0.072), substantially smaller than that of the HXF decoction (521.33 ± 28.01 nm) (Figure 1B). Furthermore, zeta potential measurements showed that NHXF possessed a higher absolute surface charge (−16.80 ± 0.96 mV) than HXF (−10.50 ± 0.90 mV) (Figure 1C), suggesting improved colloidal stability. The colloidal nature of NHXF was also supported by the observation of a distinct Tyndall effect at various concentrations of NHXF (Figure S1). In addition, transmission electron microscopy (TEM) provided direct visual evidence that NHXF consisted of uniform spherical nanoparticles, in contrast to the irregular aggregates observed in the crude HXF decoction (Figure 1D). Collectively, these findings demonstrate that nanoformulation markedly improved the physicochemical properties of HXF, yielding nanoparticles with favorable size distribution, morphology, and stability for pulmonary drug delivery.

To evaluate whether the chemical characteristics of HXF were preserved after nanoformulation, UV‐vis spectroscopy was performed and revealed a largely conserved spectral fingerprint between NHXF and the parent HXF decoction (Figure 1E). Subsequent HPLC‐MS analysis identified 24 pharmacologically active constituents in NHXF. Notably, several hydrophobic compounds relevant to RILI exhibited enhanced incorporation into the nanoformulation, indicating that NHXF effectively retained and enriched key bioactive constituents of HXF (Figure 1F; Figures S2,S3 and Table S1). Among these, neoamygdalin, hesperidin, and baicalin were detected at relatively high abundances. These constituents have been reported to exert antioxidant and anti‐inflammatory effects in tissue injury models through multiple mechanisms [27, 28, 29]. Given the chemical complexity of HXF, neoamygdalin, hesperidin, and baicalin were selected as representative bioactive markers based on their relatively high abundance in the HPLC‐MS profile to evaluate the encapsulation and release performance of the PEG‐PCL nanoformulation. The encapsulation efficiencies of neoamygdalin, hesperidin, and baicalin were 80.39%, 97.01%, and 75.10%, respectively, indicating efficient encapsulation of structurally diverse phytochemicals by PEG‐PCL. In simulated alveolar fluid, all three compounds exhibited sustained‐release profiles, with cumulative release reaching approximately 60% within 6 h and approaching a plateau after 12 h. The cumulative release rates of neoamygdalin, hesperidin, and baicalin reached 82.16 ± 3.58%, 83.67 ± 2.47%, and 86.11 ± 3.46%, respectively (Figure 1G), demonstrating the ability of NHXF to achieve controlled and prolonged release of bioactive constituents.

A major limitation of conventional herbal decoctions is their lack of tissue‐specific targeting. For pulmonary diseases, inhalation provides a direct and advantageous route for drug delivery [30, 31]. Because aerosol aerodynamic behavior critically determines pulmonary deposition efficiency, particles with aerodynamic diameters of 1–3 µm are generally considered optimal for delivery to the distal bronchioles and alveolar regions [32, 33]. Cascade impactor analysis revealed that nebulized NHXF exhibited a mass median aerodynamic diameter (MMAD) of 2.21 µm, a geometric standard deviation (GSD) of 1.52, and a fine particle fraction (FPF, < 3 µm) of 70.5%, indicating favorable aerodynamic characteristics for deep lung deposition (Figure 1H and Figure S4). Moreover, NHXF exhibited minimal changes in particle size and zeta potential after nebulization, further supporting its suitability as an inhalable nanomedicine for pulmonary delivery (Figure S5).

Capitalizing on these optimized physicochemical and aerodynamic properties, we next investigated the biodistribution of NHXF following nebulized inhalation in mice. NHXF was labeled with the near‐infrared fluorescent dye Cy5, while an equivalent dose of free Cy5 served as the control. In contrast to the free group, which was rapidly cleared and predominantly accumulated in the liver and kidneys within 3 h post‐administration, NHXF exhibited markedly enhanced pulmonary localization, with fluorescence signals remaining detectable in the lungs for up to 10 h following inhalation (Figure 1I,J). Together with its sustained‐release behavior observed in simulated alveolar fluid, these findings suggest that nanoformulation enables prolonged pulmonary residence and sustained local drug exposure after aerosol delivery. Collectively, the favorable aerodynamic properties, prolonged pulmonary exposure, and controlled‐release characteristics highlight NHXF as a promising inhalable nanoplatform for efficient pulmonary drug delivery.

### 2.2 NHXF Confers Significant Protection Against Acute Radiation‐Induced Injury in Alveolar Epithelial Cells In Vitro

**FIGURE 2:**
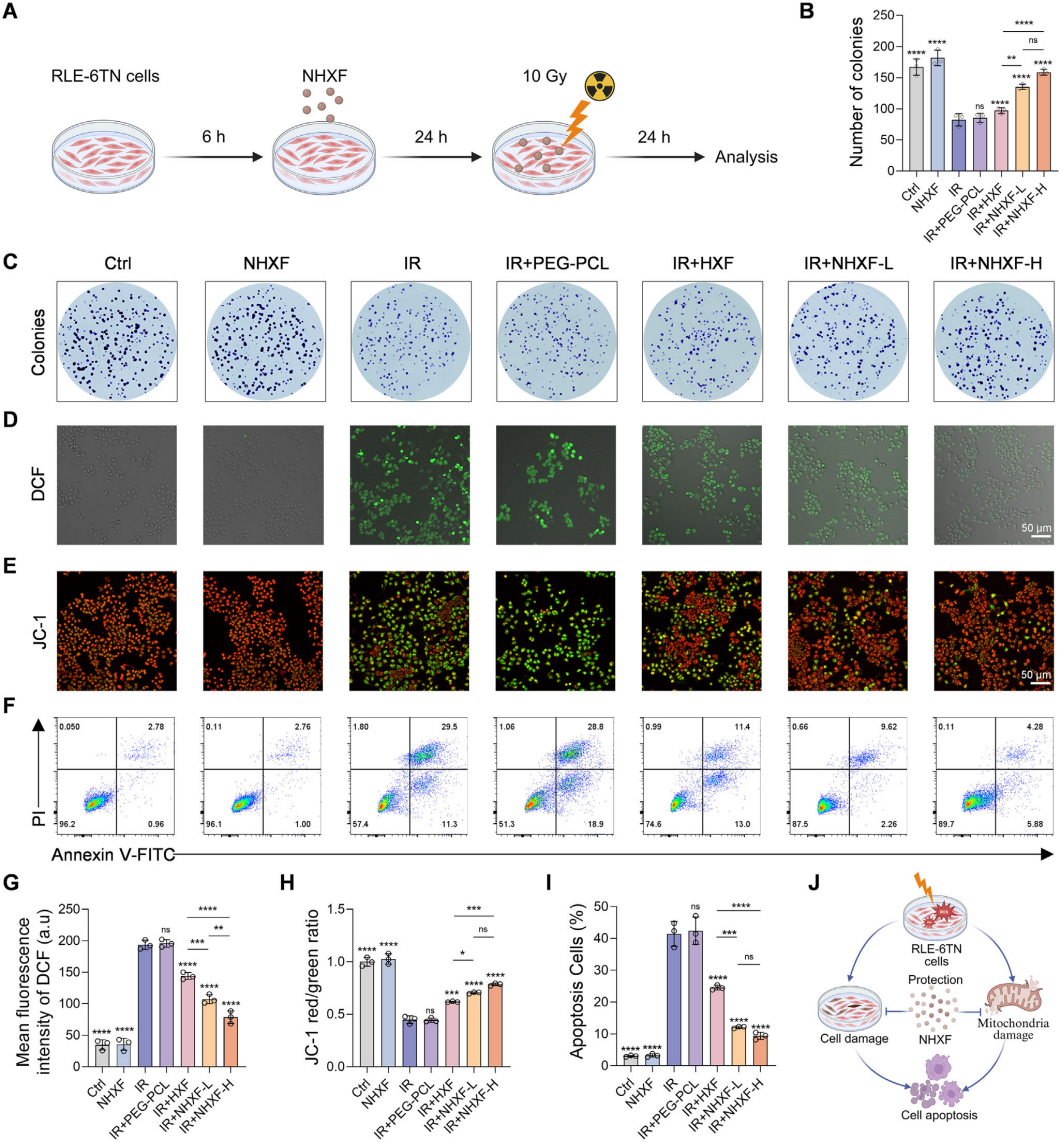
NHXF mitigates radiation‐induced injury in alveolar epithelial cells (RLE‐6TN) in vitro. (A) Schematic illustration of the experimental procedure for in vitro radioprotection studies. (B) Quantitative analysis and (C) representative images of colony formation assays. (D) Representative images of intracellular ROS levels detected using the DCFH‐DA probe (green) and (G) corresponding quantitative fluorescence intensity analysis. Scale bars, 50 µm. (E) Representative JC‐1 fluorescence images (red, JC‐1 aggregates; green, JC‐1 monomers) and (H) quantitative analysis of the red/green fluorescence intensity ratio, reflecting mitochondrial membrane potential (ΔΨm). Scale bars, 50 µm. (F) Representative flow cytometric plots of Annexin V‐FITC/PI staining and (I) quantitative analysis of apoptotic cell rates. Data in (B, G, H, and I) are presented as mean ± SEM (*n* = 3 independent experiments). Quantification of fluorescence intensity and cell counts was performed using ImageJ and FlowJo, respectively. Statistical analysis was performed using one‐way ANOVA followed by Tukey's multiple‐comparisons test. ^*^ *p* < 0.05, ^**^ *p* < 0.01, ^***^ *p* < 0.001, ^****^ *p* < 0.0001; ns, not significant. (J) Schematic illustration of the proposed mechanism by which NHXF protects against radiation‐induced alveolar epithelial cell injury.

Alveolar epithelial cells are among the primary cellular targets of ionizing radiation, and accumulating evidence indicates that the extent of epithelial injury is closely associated with the initiation and progression of pulmonary inflammation and subsequent fibrotic remodeling [34, 35, 36]. To evaluate the radioprotective effect of NHXF on alveolar epithelial cells, an in vitro radiation injury model was established using 10 Gy X‐ray irradiation (Figure 2A). CCK‐8 assays demonstrated that NHXF exhibited no significant cytotoxicity at concentrations up to 100 µg/mL and incubation times of up to 72 h, with a similar trend observed for the conventional HXF decoction (Figure S6). These findings confirm the favorable biocompatibility of NHXF. Accordingly, 20 and 100 µg/mL were selected as the low‐ and high‐dose NHXF treatment groups, respectively, for subsequent experiments. Clonogenic survival assays revealed that NHXF significantly improved post‐irradiation clonogenic survival (Figure 2B,C). Notably, both low‐ and high‐dose NHXF exhibited greater radioprotective efficacy than the conventional HXF decoction, highlighting the enhanced therapeutic benefits achieved through nanoformulation. Consistent with these findings, NHXF attenuated the transcriptional activation of DNA damage response genes, indicating reduced cellular genotoxic stress, as evidenced by the lower expression levels of DNA damage‐related genes, including *Atm*, *Tp53bp1*, *H2ax*, and *Cdkn1a* (Figure S7). Furthermore, wound‐healing assays demonstrated that NHXF markedly promoted post‐irradiation cellular recovery and migratory capacity (Figure S8).

As ionizing radiation induces excessive ROS generation, oxidative stress serves as a key driver of RILI and contributes to structural and functional damage of alveolar epithelial cells [37]. DCFH‐DA fluorescence analysis demonstrated that NHXF markedly suppressed radiation‐induced ROS accumulation in a dose‐dependent manner, with the high‐dose NHXF group exhibiting the most pronounced effect and reducing ROS levels by approximately 2.9‐fold relative to the IR group (Figure 2D,G). Consistently, JC‐1 staining demonstrated that NHXF effectively preserved mitochondrial membrane potential (ΔΨm), whereas irradiated cells exhibited substantial mitochondrial depolarization (Figure 2E,H). Furthermore, flow cytometric analysis revealed a significant reduction in Annexin V/PI‐positive apoptotic cells following NHXF treatment, indicating enhanced protection against radiation‐induced apoptosis (Figure 2F,I).

Oxidative stress, mitochondrial dysfunction, and apoptosis constitute a closely interconnected pathogenic cascade that plays a pivotal role in RILI. Following irradiation, excessive ROS generation induces oxidative damage and disrupts mitochondrial integrity, leading to mitochondrial membrane depolarization (ΔΨm loss) and metabolic dysfunction [38, 39]. The collapse of ΔΨm subsequently activates mitochondria‐dependent apoptotic pathways, resulting in extensive alveolar epithelial cell apoptosis [40]. This epithelial cell loss further amplifies inflammatory responses and tissue injury, ultimately contributing to the initiation and progression of RILI [41]. Collectively, these findings indicate that NHXF effectively attenuated this pathogenic cascade, thereby alleviating radiation‐induced injury in alveolar epithelial cells (Figure 2J). As epithelial injury represents a critical initiating event in RILI and promotes disease progression through the release of pro‐inflammatory cytokines and chemokines [42], the observed cytoprotective effects of NHXF suggest its considerable potential for mitigating RILI development.

### 2.3 Nebulized NHXF Significantly Mitigates Acute Radiation‐Induced Lung Injury in Mice

**FIGURE 3:**
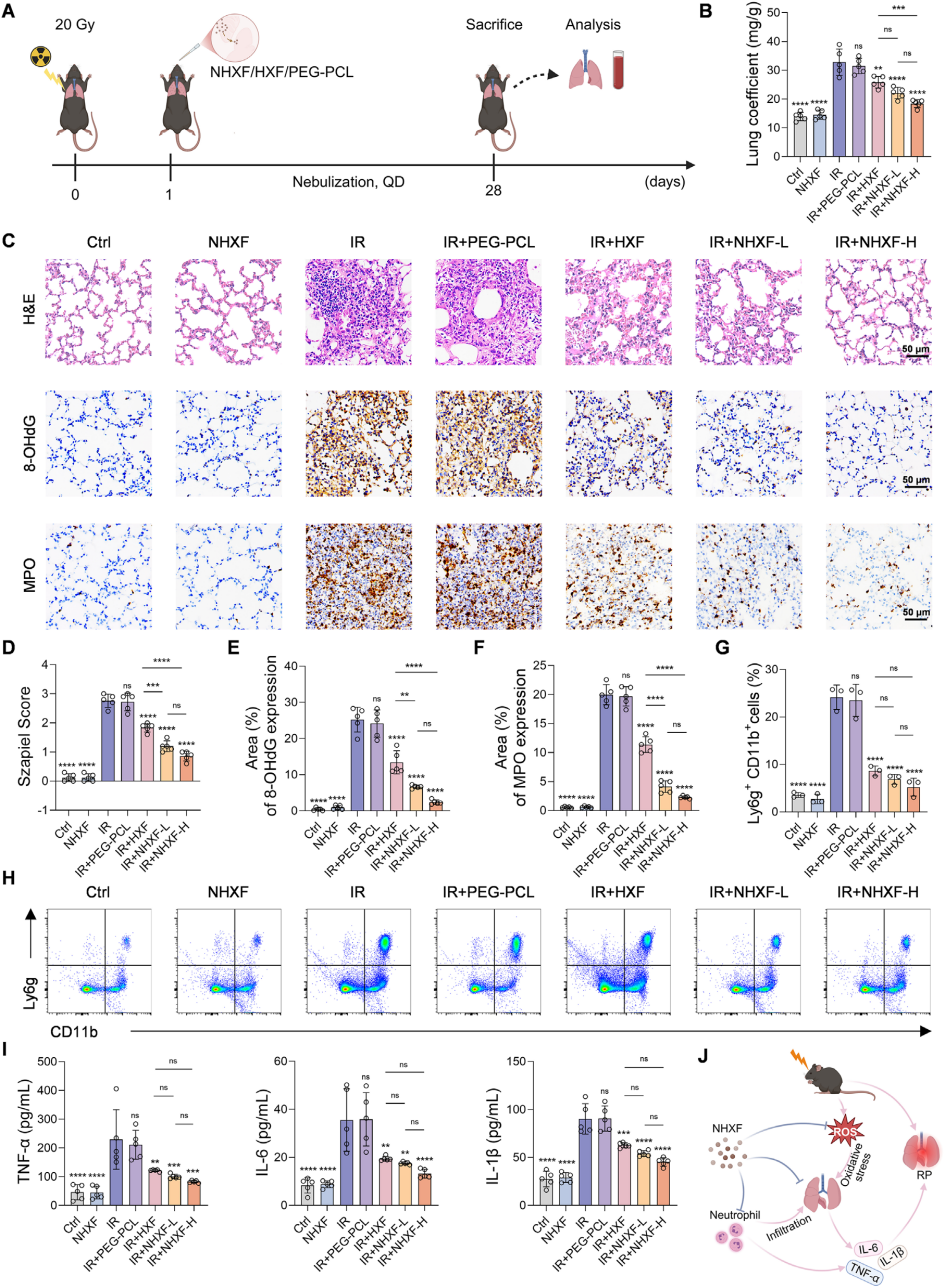
Therapeutic efficacy of nebulized NHXF in a murine model of acute RILI. (A) Schematic illustration of the in vivo experimental design. (B) Lung coefficient (lung wet weight/body weight) measured 4 weeks post‐irradiation. Data are presented as mean ± SD (*n* = 5; Hedges' *g* = 3.82; Bootstrap 95% CI for the mean difference between IR and IR+NHXF‐H (5000 resamples): 10.48 to 18.53). (C) Representative images of lung tissue sections stained with H&E and subjected to immunohistochemical staining for 8‐OHdG and MPO. Scale bars, 50 µm. (D) Szapiel scoring of lung injury based on H&E staining. Data are presented as mean ± SD (*n* = 5; Hedges' *g* = 8.52; Bootstrap 95% CI for the mean difference between IR and IR+NHXF‐H (5000 resamples): 1.65 to 2.13). (E) Quantification of 8‐OHdG‐positive areas from immunohistochemical staining. Data are presented as mean ± SD (*n* = 5; Hedges' *g* = 8.41; Bootstrap 95% CI for the mean difference between IR and IR+NHXF‐H (5000 resamples): 19.76 to 25.51). (F) Quantification of MPO‐positive areas from immunohistochemical staining. Data are presented as mean ± SD (*n* = 5; Hedges' *g* = 12.90; Bootstrap 95% CI for the mean difference between IR and IR+NHXF‐H (5000 resamples): 16.30 to 19.25). (G) Quantitative analysis of CD11b^+^Ly6G^+^ neutrophils in lung single‐cell suspensions and (H) the representative flow cytometric plots. (I) Serum concentrations of the pro‐inflammatory cytokines TNF‐α, IL‐6, and IL‐1β measured by ELISA. Data in (G) and (I) are presented as mean ± SD (*n* = 5 mice per group). Quantification of immunohistochemical staining was performed using ImageJ, histological scoring was conducted in a blinded manner, and cell counts were analyzed using FlowJo. Statistical analysis was performed using one‐way ANOVA followed by Tukey's multiple‐comparisons test. ^*^ *p* < 0.05, ^**^ *p* < 0.01, ^***^ *p* < 0.001, ^****^ *p* < 0.0001; ns, not significant. (J) Schematic illustration of the proposed in vivo protective mechanism of NHXF.

To evaluate the in vivo therapeutic efficacy of NHXF against acute RILI, a mouse model was established by delivering a single 20 Gy dose of X‐ray irradiation to the right thoracic region. Mice subsequently received NHXF daily via nebulized inhalation until 28 days post‐irradiation (Figure 3A). Longitudinal monitoring of body weight indicated that the IR+NHXF‐H group exhibited a body‐weight profile more closely resembling that of the Ctrl and NHXF‐alone groups, in contrast to the progressive weight loss observed in the IR group (Figure S9). At day 28 post‐irradiation, the lung coefficient, an indicator of pulmonary injury severity, was markedly elevated in irradiated animals compared with controls, whereas nebulized NHXF significantly attenuated this increase (Figure 3B). To further assess structural lung damage, histopathological analysis was performed. Hematoxylin and eosin (H&E) staining revealed severe alveolar destruction, interstitial thickening, and prominent inflammatory cell infiltration in the irradiated group, whereas these pathological alterations were substantially alleviated in the high‐dose NHXF group, accompanied by preservation of alveolar architecture and a reduced Szapiel lung injury score (Figure 3C,D). Immunohistochemical staining for 8‐OHdG, a biomarker of oxidative DNA damage, revealed a substantial increase in positive staining in the IR group, which was markedly reduced by NHXF treatment (25.8% vs. 2.4%), indicating that NHXF significantly attenuated radiation‐induced oxidative DNA damage in vivo (Figure 3C,E).

Beyond epithelial and structural injury, the acute phase of RILI is frequently accompanied by the development of RP [43]. Analysis of bronchoalveolar lavage fluid (BALF) revealed an increased total cell count in the IR group, with numerous enlarged and activated inflammatory cells detected by Diff‐Quik staining (Figure S10). Neutrophils are among the earliest and most abundant inflammatory cells recruited following RILI. Radiation‐triggered oxidative stress activates inflammatory signaling cascades and upregulates chemokine expression, thereby promoting neutrophil infiltration and amplifying tissue damage through the release of pro‐inflammatory mediators [44]. Accordingly, neutrophil accumulation in lung tissue was further evaluated. At the transcriptional level, chemokines associated with neutrophil recruitment, including *Cxcl1*, *Cxcl2*, and *Cxcl5*, were markedly upregulated in both lung tissue and epithelial cells in the irradiated group, whereas NHXF treatment significantly suppressed their expression compared with the IR group (Figure S11). Consistently, immunohistochemical staining for the neutrophil marker MPO confirmed a pronounced reduction in MPO‐positive areas in the IR+NHXF‐H group compared with the IR group (20.0% vs. 2.3%), whereas relatively strong MPO positivity persisted in the IR+HXF group (Figure 3C,F). Complementing these histological findings, flow cytometry demonstrated that NHXF treatment significantly decreased the proportion of neutrophils in both lung tissue and peripheral blood relative to the IR group (Figure 3G,H and Figure S12).

To further characterize radiation‐induced inflammatory responses, cytokine levels in serum and BALF were quantified by ELISA. Pro‐inflammatory mediators, including TNF‐α, IL‐6, IL‐1β, TGF‐β1, MCP‐1, and IFN‐γ, were markedly elevated in the irradiated group but significantly reduced following high‐dose NHXF treatment. In contrast, the anti‐inflammatory cytokine IL‐10 was increased in NHXF‐treated animals (Figure 3I and Figures S13,S14). Collectively, these findings indicate that NHXF mitigates radiation‐induced inflammatory responses by rebalancing the inflammatory cytokine milieu. Together with the observed reductions in epithelial injury and neutrophil infiltration, these results support a model in which NHXF interrupts the reciprocal reinforcement between tissue injury and inflammation, thereby conferring effective protection against radiation‐induced pulmonary damage (Figure 3J).

To evaluate the systemic safety of NHXF, comprehensive hematological and serum biochemical analyses were performed in addition to body weight monitoring. Complete blood count analysis showed that all NHXF‐treated groups maintained hematological parameters within physiological reference ranges (Figure S15). In contrast, the irradiated group exhibited marked abnormalities in leukocyte and neutrophil counts, likely reflecting radiation‐induced myelosuppression and systemic inflammatory responses, respectively. Serum biochemical assessments, including liver, renal, and cardiac function indices, revealed no evidence of organ dysfunction in any NHXF‐treated animals (Figure S16). Consistently, histopathological examination of major organs (heart, liver, spleen, and kidney) showed no detectable structural abnormalities across all groups (Figure S17). These findings are consistent with previously reported safety data for HXF [9, 10], and indicate that nanoformulation preserves the favorable biosafety profile of the parent formulation.

To determine whether the radioprotective effects of NHXF compromise the antitumor efficacy of radiotherapy, both in vitro and in vivo tumor models were evaluated. In lung cancer cell lines (LLC and A549), irradiation significantly suppressed cell viability and clonogenic capacity. Notably, concomitant NHXF treatment did not attenuate the cytotoxic or clonogenic inhibitory effects of irradiation in either model (Figure S18). These findings were further validated in a murine subcutaneous lung tumor model. Conventional fractionated radiotherapy (3 × 7 Gy, administered every other day) induced pronounced tumor regression and growth inhibition (Figure S19A). Importantly, concomitant treatment with NHXF via peritumoral injection and inhalation did not alter the therapeutic efficacy of radiotherapy, as comparable tumor growth suppression was observed between the IR and IR+NHXF groups (Figure S19B,C,E). Histopathological analysis further confirmed extensive tumor structural disruption and reduced Ki67 expression following irradiation, with no detectable differences between irradiated tumors treated with or without NHXF (Figure S19D,F,G). In addition, NHXF alone exerted negligible effects on tumor growth and proliferation. Compared with normal tissues, tumors are characterized by poor differentiation and a highly disordered microenvironment. Consequently, the biological processes underlying radiation‐induced tumor suppression may be fundamentally distinct from those driving normal tissue injury, rendering tumors less responsive to protective interventions and thereby allowing NHXF to alleviate normal tissue toxicity without compromising the antitumor efficacy of radiotherapy [45]. Collectively, these results demonstrate that NHXF selectively protects normal alveolar epithelial cells from radiation‐induced injury while preserving tumor radiosensitivity, supporting its potential as an adjuvant protective strategy during radiotherapy.

While nebulization enhances local drug delivery and retention within the lungs, direct nebulization of traditional herbal decoctions is often limited by low delivery efficiency and potential microbial contamination [46]. Our study provides proof‐of‐concept for an innovative drug development paradigm that integrates the multi‐component pharmacology of TCM with nanotechnology‐based drug delivery. The results demonstrate that nebulized NHXF significantly ameliorates radiation‐induced oxidative stress, reduces neutrophil recruitment, and suppresses local pro‐inflammatory cytokine production, thereby effectively alleviating pulmonary inflammation and tissue injury. Crucially, the blank nanocarrier control (PEG‐PCL) conferred no therapeutic benefit, confirming that the observed therapeutic effects are attributable to the bioactive constituents of HXF, while the nanocarrier functions primarily as a delivery platform that enhances pulmonary targeting and retention.

### 2.4 NHXF Exhibits Favorable Potential for RILI Management, Relative to Dexamethasone

Glucocorticoids remain a guideline‐recommended symptomatic therapy for RILI due to their well‐established anti‐inflammatory effects [47]. However, their clinical utility is often limited by dose‐dependent adverse effects and disease relapse following treatment withdrawal, representing a major therapeutic challenge [3]. This limitation is thought to arise, at least in part, from persistent radiation‐induced tissue injury, whereby damaged cells continuously release chemokines that recruit inflammatory cells. Consequently, anti‐inflammatory monotherapy may be insufficient to address the underlying pathological drivers of RILI [6, 48]. Given its ability to protect alveolar epithelial cells from radiation‐induced injury, we next investigated whether NHXF could overcome the limitations associated with glucocorticoid therapy.

**FIGURE 4:**
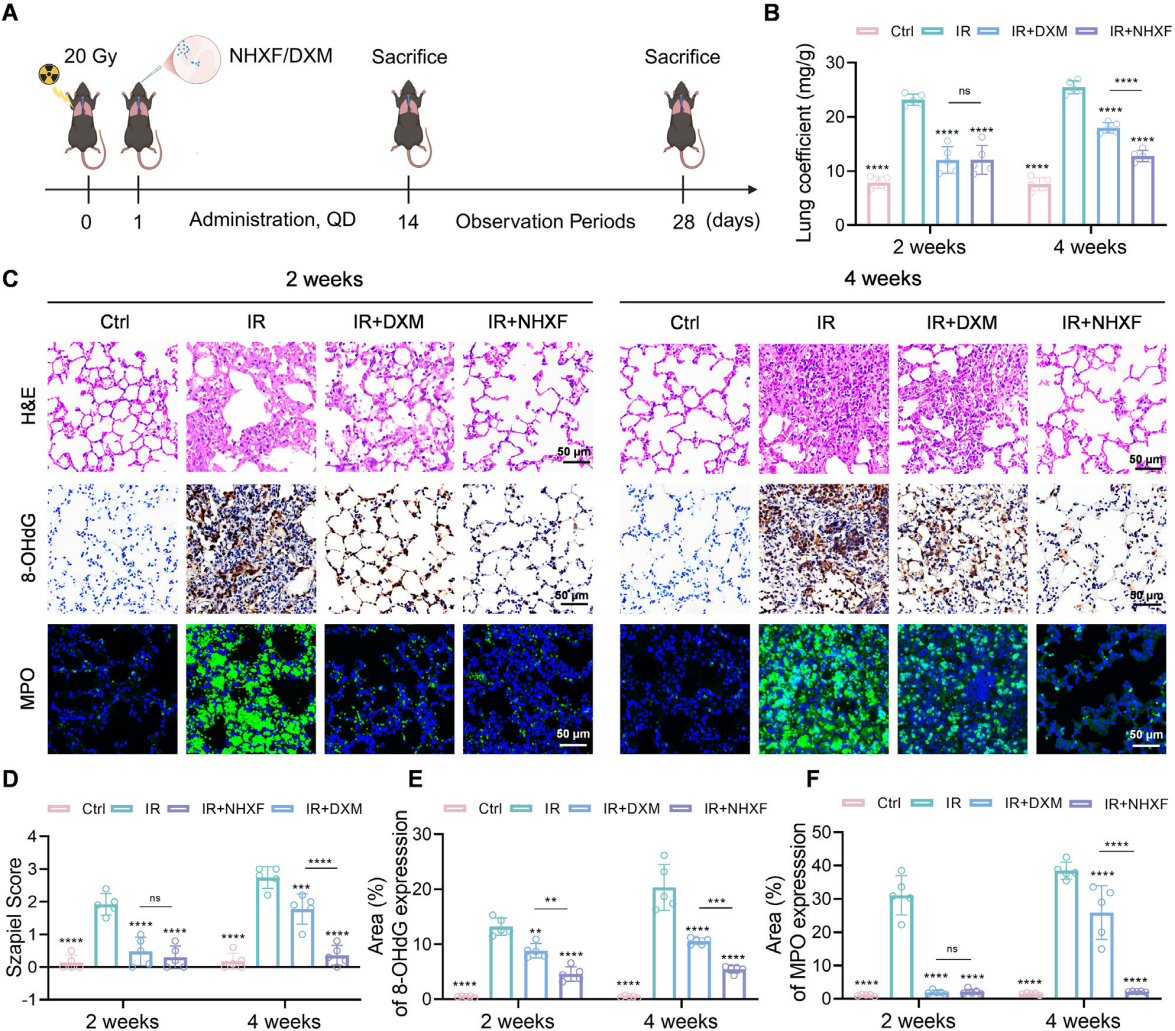
Nebulized NHXF provides favorable potential against RILI compared with dexamethasone (DXM). (A) Schematic diagram of the experimental design for the comparative study with DXM. (B) Lung coefficient measured at 2 and 4 weeks post‐irradiation. (C) Representative images of lung tissue sections showing H&E staining, 8‐OHdG immunohistochemistry (IHC), and MPO immunofluorescence (IF) at 2 and 4 weeks. Scale bars, 50 µm. (D) Szapiel scoring based on H&E staining. (E) Quantification of 8‐OHdG‐positive areas. (F) Quantification of MPO‐positive areas. Data in (B, D–F) are presented as mean ± SD (*n* = 5 mice per group). Quantification of 8‐OHdG‐positive and MPO‐positive areas was performed using ImageJ. Statistical analysis was performed using one‐way ANOVA followed by Tukey's multiple‐comparisons test. ^*^ *p* < 0.05, ^**^ *p* < 0.01, ^***^ *p* < 0.001, ^****^ *p* < 0.0001; ns, not significant.

Mice received short‑term treatment with either NHXF or DXM, followed by a drug withdrawal period (Figure 4A). During the initial treatment phase, both NHXF and DXM effectively reduced the lung coefficient and preserved alveolar architecture. However, following treatment withdrawal, the IR+DXM group exhibited a pronounced rebound in injury parameters, whereas NHXF maintained protective effects (Figure 4B–D). Consistently, 8‐OHdG staining revealed a larger positive area in the DXM group than in the NHXF group at 2 weeks post‐irradiation, and this difference became more pronounced by week 4 due to a further increase in oxidative DNA damage in the DXM group (Figure 4C,E). MPO immunostaining similarly demonstrated divergent effects on pulmonary inflammation: although both DXM and NHXF reduced neutrophil infiltration at the early stage, MPO positivity rebounded after DXM withdrawal, whereas NHXF maintained significantly lower MPO levels throughout the observation period (Figure 4C,F). Collectively, these findings suggest that NHXF, by protecting alveolar epithelial cells from radiation‐induced injury, targets an upstream pathological process rather than merely suppressing inflammatory responses in lung tissue. Consequently, NHXF may represent a promising strategy for early intervention in RILI, either as monotherapy or as an adjunct to glucocorticoids, with the potential to achieve more durable therapeutic benefits and reduced rebound inflammation after treatment withdrawal. Future studies are warranted to optimize the dosing regimen and to evaluate potential combination regimens in clinically relevant models.

### 2.5 Transcriptomic Profiling Elucidates the Protective Mechanism of NHXF Against Acute RILI

**FIGURE 5:**
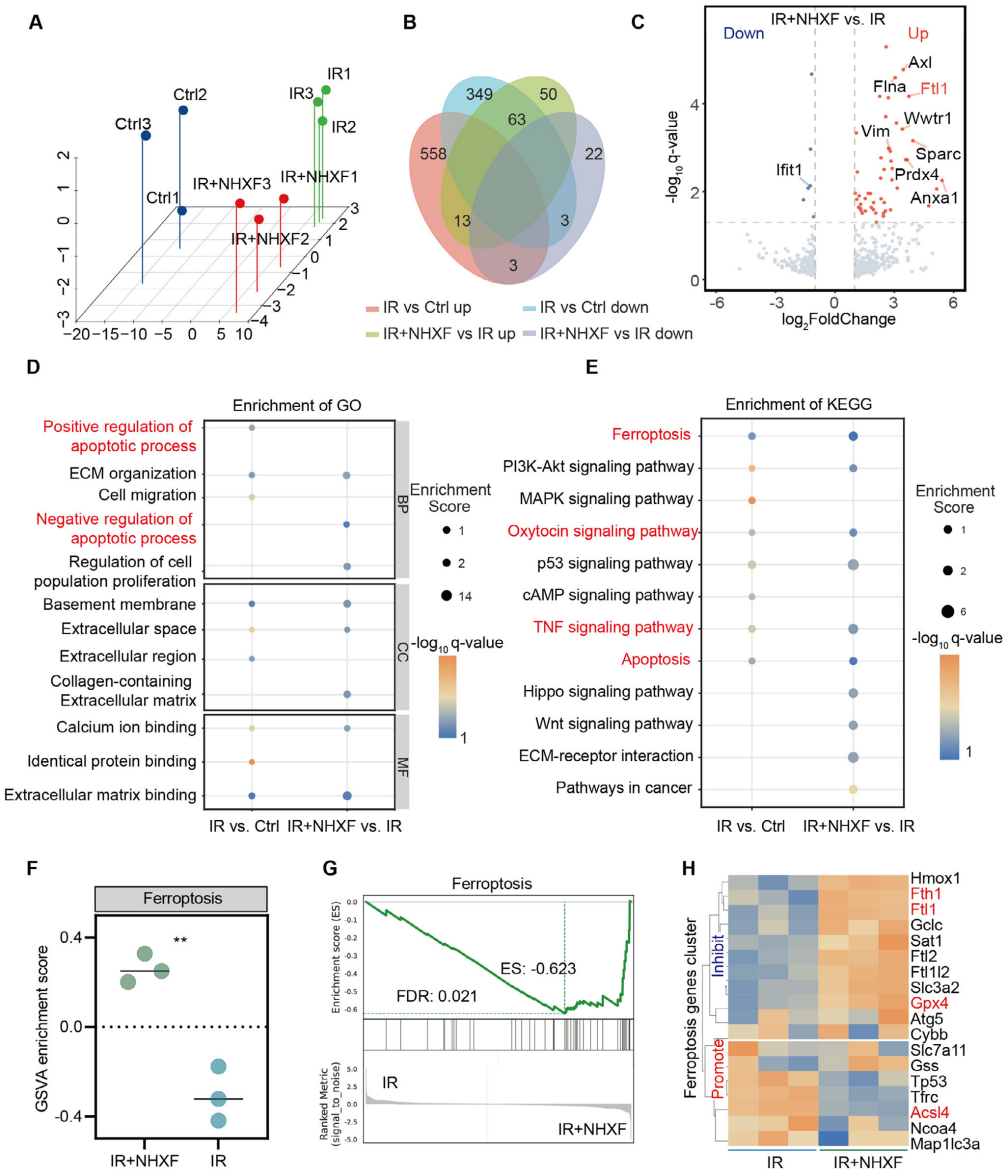
Transcriptomic analysis identifies ferroptosis as a key pathway modulated by NHXF. (A) Principal component analysis (PCA) of RNA‐seq data from RLE‐6TN cells in the Ctrl, IR, and IR+NHXF groups (*n* = 3 biologically independent samples per group). (B) Venn diagram showing the overlap of differentially expressed genes (DEGs). (C) Volcano plot of DEGs in the IR+NHXF versus IR comparison (|log_2_FC| > 1, FDR‐adjusted *q* < 0.05). (D, E) Bubble plots of significantly enriched Gene Ontology (GO) terms and Kyoto Encyclopedia of Genes and Genomes (KEGG) pathways from the IR versus Ctrl and IR+NHXF versus IR comparisons. (F) Gene Set Variation Analysis (GSVA) enrichment scores for the ferroptosis pathway. (G) Gene Set Enrichment Analysis (GSEA) plot of the ferroptosis pathway. NES, normalized enrichment score; FDR, false discovery rate. (H) Heatmap of normalized expression values for key ferroptosis‐related genes.

To systematically investigate the molecular mechanisms underlying the protective effects of NHXF against acute RILI, RNA sequencing was performed on alveolar epithelial cells. Principal component analysis (PCA) revealed a clear separation between the IR+NHXF and IR groups, indicating substantial reprogramming of the transcriptional landscape following NHXF intervention (Figure 5A). A total of 82 overlapping differentially expressed genes (DEGs) were identified between the IR vs. control and IR+NHXF vs. IR comparisons, suggesting a broad multi‐target regulatory profile of NHXF (Figure 5B). Volcano plot analysis highlighted key DEGs across the two comparisons, including *Ftl1*, *Vim*, *Flna*, and *Axl*, which exhibited significant differential expression and opposite regulatory trends across the two comparisons (Figure 5C and Figure S20). Among these, ferritin light chain 1 (*Ftl1*), which forms the ferritin iron‐storage complex together with ferritin heavy chain 1 (*Fth1*), plays an essential role in maintaining iron homeostasis and regulating ferroptosis [49]. Protein‐protein interaction (PPI) network analysis indicated that *Ftl1* is closely connected with canonical ferroptosis‐related regulators such as *Gpx4* and *Acsl4*, suggesting that ferroptosis modulation may represent a central mechanism underlying the protective effects of NHXF (Figure S21).

Bioinformatic enrichment analysis was conducted to clarify the involvement of ferroptosis‐related pathways. GO and KEGG analyses revealed that DEGs were predominantly associated with apoptosis regulation, the TNF signaling pathway, the oxytocin signaling pathway, and ferroptosis (Figure 5D,E), which have well‐documented associations with the pathogenesis of lung injury [50, 51]. In addition, the oxytocin signaling pathway has recently been implicated in protection against RILI [52], further supporting the relevance of this pathway to our model. Gene Set Variation Analysis (GSVA) further demonstrated that NHXF markedly reprogrammed ferroptosis‐related gene signatures (Figure 5F). Consistently, Gene Set Enrichment Analysis (GSEA) confirmed significant modulation of the ferroptosis pathway in the IR+NHXF group compared with the IR group (FDR = 0.021) (Figure 5G). Accordingly, heatmap analysis of ferroptosis‐associated genes revealed coordinated downregulation of pro‐ferroptotic regulators (e.g., *Acsl4* and *Tfrc*) and upregulation of ferroptosis‐related protective genes (e.g., *Slc7a11*, *Gpx4*, *Fth1*, and *Ftl1*) following NHXF treatment (Figure 5H). Accumulating evidence indicates that ferroptosis, an iron‐dependent form of lipid peroxidation‐driven regulated cell death, contributes to the pathogenesis of radiation‐induced injury across multiple tissues [53]. Our transcriptomic and enrichment analyses suggest that NHXF exerts multifaceted protective effects against RILI, with ferroptosis modulation contributing to its protective activity against radiation‐induced injury.

### 2.6 NHXF Modulates Cellular Iron Homeostasis and Suppresses Ferroptosis in Epithelial Cells

**FIGURE 6:**
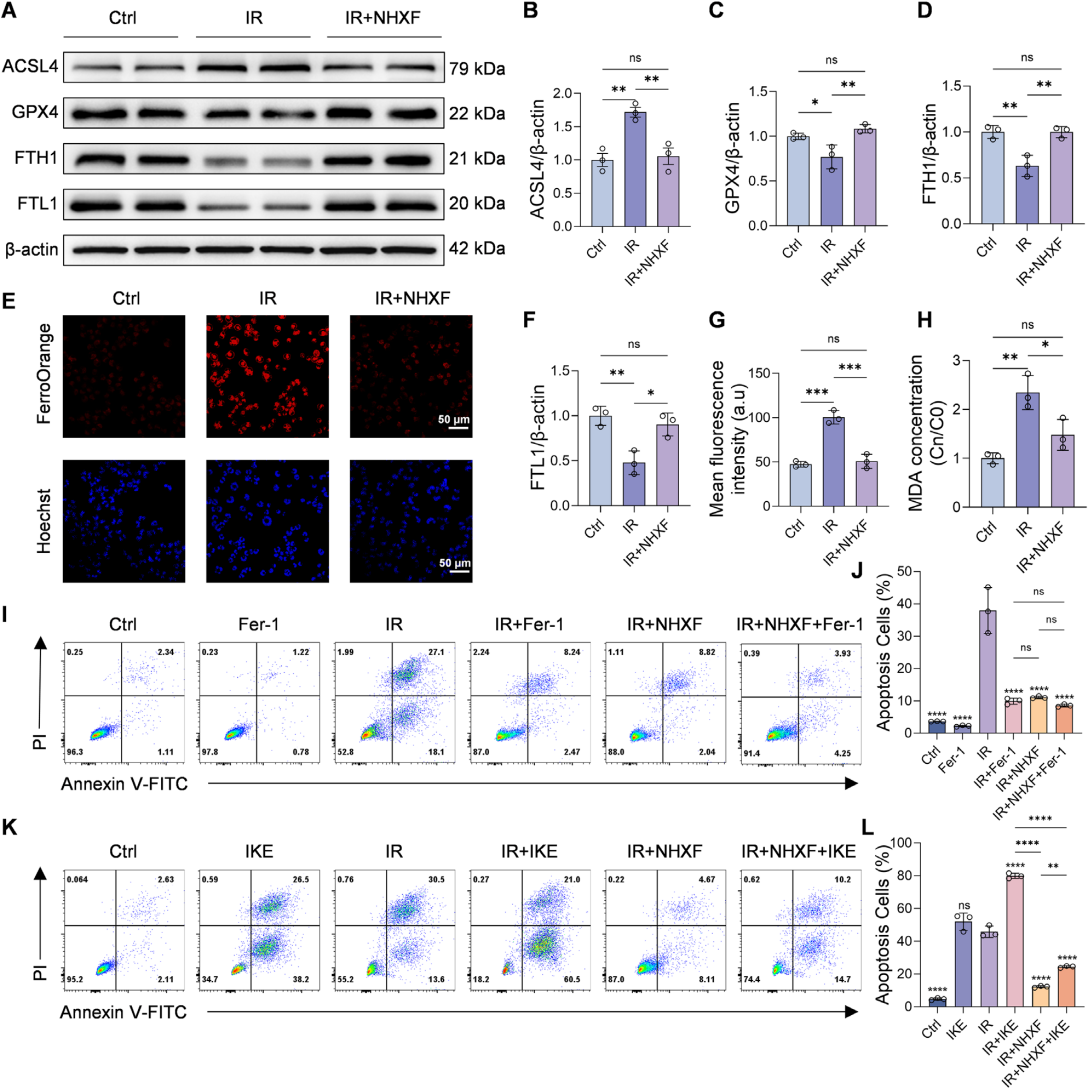
NHXF suppresses radiation‐induced ferroptosis in alveolar epithelial cells. (A) Representative Western blot images and (B–D, F) quantitative analysis of ferroptosis‐related proteins (ACSL4, GPX4, FTH1, and FTL1). β‐actin was used as the loading control. (E) Representative fluorescence images of intracellular labile Fe^2^^+^ detected by FerroOrange staining and (G) corresponding quantitative analysis of fluorescence intensity. Scale bars, 50 µm. (H) Normalized intracellular malondialdehyde (MDA) levels (Cn/C0). (I) Representative flow cytometry scatter plots of apoptosis detected by Annexin V‐FITC/PI staining following treatment with the ferroptosis inhibitor ferrostatin‐1 (Fer‐1), and (J) corresponding quantitative analysis of apoptotic cell rates. (K) Representative flow cytometry scatter plots of apoptosis detected by Annexin V‐FITC/PI staining following treatment with the ferroptosis inducer IKE, and (L) corresponding quantitative analysis of apoptotic cell rates. Data in (B–E, G, H, J, and L) are presented as mean ± SEM (*n* = 3 independent experiments). Quantification of Western blot bands and fluorescence intensity was performed using ImageJ, and cell counts were analyzed using FlowJo. Statistical analysis was performed using one‐way ANOVA followed by Tukey's multiple‐comparisons test. ^*^ *p* < 0.05, ^**^ *p* < 0.01, ^***^ *p* < 0.001, ^****^ *p* < 0.0001; ns, not significant.

To further investigate the involvement of ferroptosis‐related regulation suggested by transcriptomic analysis, representative ferroptosis‐associated proteins were examined. Radiation markedly downregulated anti‐ferroptotic proteins, including FTL1, FTH1, and GPX4, while increasing the expression of the pro‐ferroptotic regulator ACSL4. In contrast, NHXF treatment significantly reversed these alterations and partially restored the expression profile of ferroptosis‐related regulators in irradiated cells (Figure 6A–D,F). Ftl1 and Fth1 are critical iron‐storage proteins that sequester excess ferrous iron (Fe^2^^+^) and maintain intracellular iron homeostasis [54]. Consistently, FerroOrange staining revealed a significant reduction in labile iron accumulation in the IR+NHXF group compared with the IR group, suggesting that NHXF enhances iron sequestration capacity and mitigates iron overload (Figure 6E,G).

In addition, GPX4 and ACSL4 constitute a functional antagonistic axis in the regulation of lipid peroxidation. ACSL4 facilitates the incorporation of polyunsaturated fatty acids into membrane phospholipids, thereby providing essential substrates for lipid peroxidation, whereas GPX4 detoxifies lipid hydroperoxides and prevents their accumulation [55, 56]. Accordingly, intracellular malondialdehyde (MDA) levels were measured as a representative marker of lipid peroxidation. Radiation exposure significantly increased MDA accumulation, whereas NHXF treatment markedly attenuated this effect (Figure 6H). In summary, FTL1/FTH1 and GPX4/ACSL4 represent important regulatory components associated with ferroptosis, contributing to the maintenance of iron homeostasis and regulation of lipid peroxidation, respectively. Our findings suggest that NHXF simultaneously preserves iron homeostasis and limits lipid peroxidation, thereby suppressing ferroptosis and protecting irradiated alveolar epithelial cells from radiation‐induced injury.

To further establish the functional involvement of ferroptosis in the protective effects of NHXF, the ferroptosis inhibitor ferrostatin‐1 (Fer‐1) and the ferroptosis inducer imidazole ketone erastin (IKE) were applied in vitro [57, 58], and their effects on intracellular ROS levels and apoptosis were evaluated. Fer‐1 alone significantly attenuated radiation‐induced oxidative stress and apoptotic cell death in alveolar epithelial cells. In contrast, IKE markedly abolished the cytoprotective effects of NHXF and further exacerbated ROS accumulation and apoptosis in the IR+IKE group (Figure 6I–L and Figure S22). These findings provide functional evidence that inhibition of ferroptosis is an important contributor to the radioprotective effects of NHXF in alveolar epithelial cells.

### 2.7 Limitations & Future Directions

Despite these encouraging findings, several limitations should be acknowledged. First, owing to the inherent compositional complexity of NHXF, comprehensive characterization of the nanoformulation, particularly the loading characteristics of its major bioactive constituents and its pharmacokinetic (PK) behavior, remains challenging. Future studies will focus on the systematic characterization and PK profiling of multiple major bioactive constituents within NHXF to better define its in vivo fate. In addition, although NHXF demonstrated significant protection against acute RILI, a more comprehensive evaluation is warranted, including extended observation periods (20–30 weeks post‐irradiation), pulmonary function assessments, and larger sample sizes to provide more robust support for our findings. Moreover, as the first study to propose nebulized NHXF as a preventive and therapeutic strategy for RILI, additional mechanistic investigations are warranted to provide more direct evidence. These studies should include analyses of radiation‐induced DNA damage responses, characterization of the roles and interactions among multiple pulmonary cell populations (e.g., epithelial cells, endothelial cells, inflammatory cells, and fibroblasts), as well as deeper exploration of ferroptosis‐related mechanisms through approaches such as direct lipid peroxidation assays (e.g., C11‐BODIPY staining) and genetic interventions targeting ferroptosis‐related regulators (e.g., shFTL1 and shGPX4). These efforts will further elucidate the pharmacological basis of NHXF and facilitate its future clinical translation.

## 3 Conclusion

**FIGURE 7:**
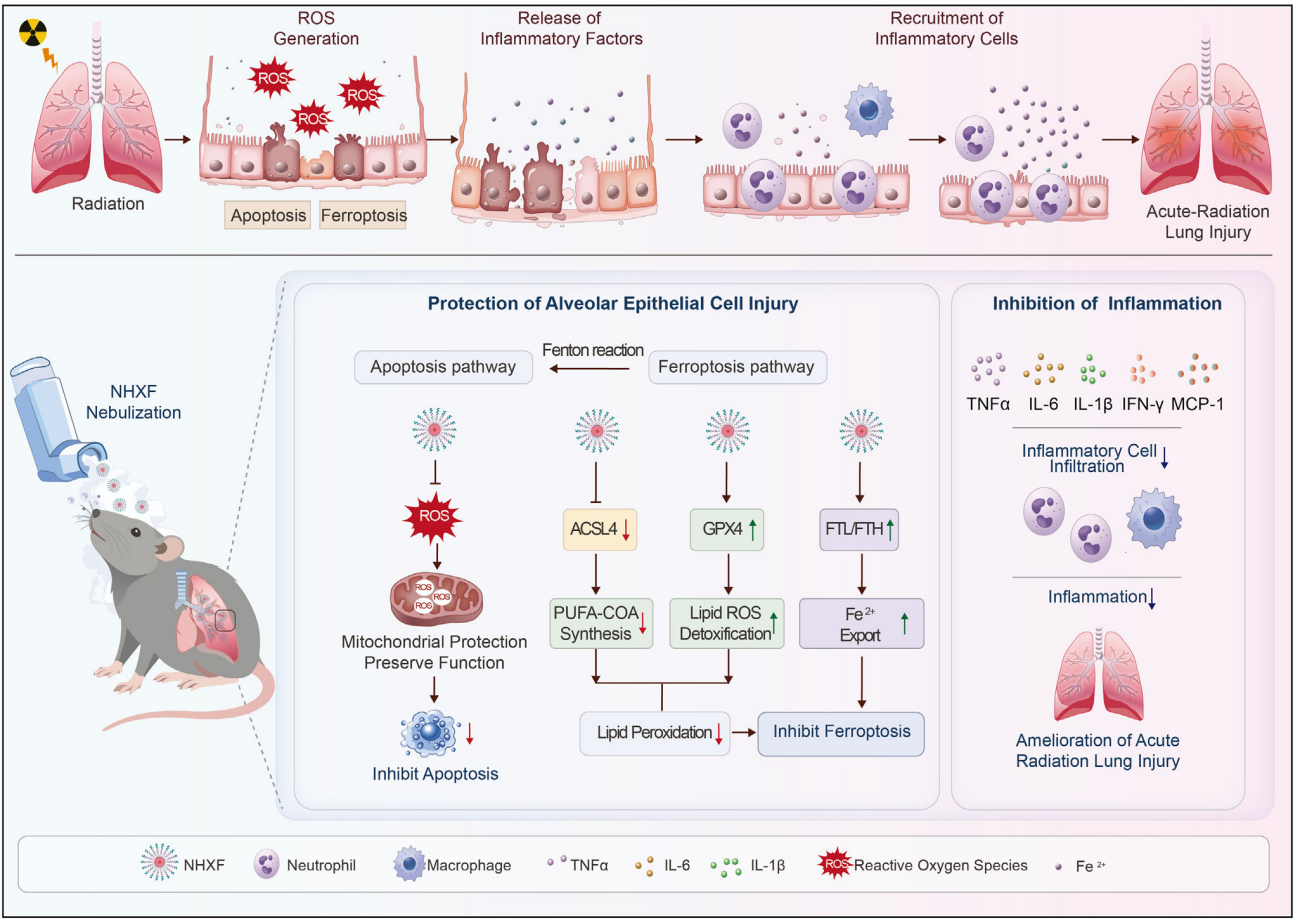
Proposed mechanism underlying the protective effects of NHXF against acute RILI. Radiation exposure triggers excessive ROS generation in alveolar epithelial cells, leading to primary epithelial injury through the induction of apoptosis and ferroptosis. Damaged epithelial cells subsequently release pro‐inflammatory mediators that recruit inflammatory cells and amplify local inflammatory responses, thereby driving the progression of acute RILI. NHXF alleviates acute RILI through two complementary protective mechanisms. First, NHXF protects alveolar epithelial cells by suppressing ROS accumulation and apoptosis. Simultaneously, NHXF attenuates ferroptosis through the dual regulation of lipid peroxidation and iron homeostasis, as evidenced by modulation of the ACSL4/GPX4 axis and enhancement of FTL1/FTH1‐mediated iron sequestration. Given the close interplay between ferroptosis and apoptosis, inhibition of ferroptosis may further mitigate apoptosis‐associated epithelial damage. Second, NHXF suppresses the release of pro‐inflammatory cytokines, thereby reducing inflammatory cell infiltration and attenuating acute pulmonary inflammation. Through the coordinated inhibition of epithelial cell death and inflammatory responses, NHXF effectively ameliorates acute RILI.

In summary, this study demonstrates that the development of the nanoformulation NHXF from HXF represents a robust and promising strategy for the prevention and treatment of acute RILI. Through a rational PEG‐PCL‐based self‐assembly approach, we effectively addressed the key pharmaceutical limitations of the conventional decoction, resulting in a nanomicellar system with optimized pulmonary delivery and retention properties. Our results indicate that NHXF acts locally at sites of lung injury to protect alveolar epithelial cells from radiation‐induced damage through coordinated antioxidative and anti‐ferroptotic mechanisms. Furthermore, by preserving epithelial integrity, NHXF attenuates subsequent inflammatory responses in lung tissue (Figure 7). Notably, this anti‐inflammatory action appears to operate at a more upstream regulatory level, which may support the potential to establish a novel RILI management strategy. Collectively, these findings provide a strong rationale for the further development and clinical translation of NHXF and highlight the potential of integrating traditional medicine with modern nanotechnology for the treatment of radiation‐induced tissue injury.

## 4 Experimental Section

### 4.1 Preparation of NHXF

The composition of Huaxian Formula (HXF) is provided in Supplementary Table S2. The herbal mixture was soaked in distilled water (1000 mL) for 30 min, boiled for 30 min, and centrifuged at 8000 rpm for 30 min. The resulting supernatant was lyophilized to obtain HXF powder. NHXF micelles were prepared by the solvent evaporation method. Briefly, HXF powder (10 mg) and PEG5000‐PCL10000 (10 mg; Xi'an Qiyue, China) were dissolved in tetrahydrofuran (10 mL; Merck, Germany) and added dropwise to ultrapure water (10 mL) under stirring at 300 rpm. The suspension was sonicated for 10 min and then subjected to solvent evaporation overnight at 4°C with stirring at 200 rpm. The resulting NHXF micelles were lyophilized and stored at −80°C.

### 4.2 In Vivo Biodistribution of NHXF

NHXF micelles were prepared as described above, except that PEG‐PCL was replaced with Cy5‐conjugated PEG‐PCL to yield HXF‐PEG‐PCL‐Cy5. Mice received continuous nebulized inhalation of HXF‐PEG‐PCL‐Cy5 (2 mL, 1 mg/mL) or an equivalent dose of Cy5‐free micelles (2 mL, 25 µg/mL). At 10 min and 3, 6, and 10 h post‐inhalation, mice were anesthetized and euthanized, and major organs, including the heart, liver, spleen, lungs, and kidneys, were harvested for fluorescence imaging to assess the biodistribution of Cy5‐labeled NHXF.

### 4.3 Cell Culture

Rat type II alveolar epithelial cells (RLE‐6TN) were obtained from VetCell Biological Technology Co., Ltd. (China), while murine Lewis lung carcinoma (LLC) cells and human lung adenocarcinoma (A549) cells were obtained from the Cell Bank of the Chinese Academy of Sciences (China). All cell lines were cultured in DMEM supplemented with 10% fetal bovine serum and 1% penicillin‐streptomycin and maintained at 37°C in a humidified atmosphere containing 5% CO_2_. The culture medium was replaced every 2–3 days, and cells were passaged at a ratio of 1:3 upon reaching 70%–80% confluence.

### 4.4 Detection of Intracellular Reactive Oxygen Species (ROS)

Cells were seeded in 48‐well plates at 1 × 10^4^ cells/well and treated as indicated, followed by 10 Gy X‐ray irradiation. Thirty minutes post‐irradiation, cells were incubated with 1 µM DCFH‐DA (Beyotime, China) at 37°C for 30 min. DCFH‐DA passively enters cells, is hydrolyzed by intracellular esterases to DCFH, which is oxidized by ROS to fluorescent DCF. Intracellular green fluorescence was imaged using confocal laser scanning microscopy (CLSM; LSM 880, Carl Zeiss), and mean fluorescence intensity was quantified with ImageJ to assess ROS levels.

### 4.5 Measurement of Mitochondrial Membrane Potential (ΔΨm)

At 24 h post‐treatment and irradiation, cells were incubated with JC‐1 (Solarbio, China) at 37°C for 30 min. JC‐1 forms red‐fluorescent aggregates in mitochondria with high membrane potential and remains as green‐fluorescent monomers in cells with low potential. Red and green fluorescence were imaged using confocal laser scanning microscopy, and the red/green fluorescence ratio was quantified with ImageJ to assess ΔΨm changes.

### 4.6 Analysis of Cellular Apoptosis

Cells were seeded in 6‐well plates at 1 × 10^5^ cells per well. 24 h after treatment, adherent and floating cells were collected, washed with PBS, and resuspended in 1× binding buffer (100 µL). Samples were incubated with Annexin V‐FITC (5 µL; 4abio, China) in the dark for 5 min, followed by the addition of propidium iodide (PI) and quenching with PBS (400 µL). Apoptosis was analyzed by spectral flow cytometry (Cytek Aurora/Northern Lights; Cytek Biosciences, USA). Annexin V^+^ PI^−^ cells were classified as early apoptotic, whereas Annexin V^+^ PI^+^ cells were considered late apoptotic or necrotic. Data were analyzed using FlowJo.

### 4.7 Animals

Eighty‐four male C57BL/6J mice (8–10 weeks) were purchased from Beijing HFK Bioscience Co., Ltd. and housed under SPF conditions with a 12:12 h light/dark cycle at 22°C, with ad libitum access to food and water. Mice were acclimated for one week prior to experiments. All procedures were approved by the Animal Ethics Committee of Sichuan Cancer Hospital (Approval No.: SCCHEC‐04‐2023‐023) and conducted in accordance with institutional and legal guidelines.

### 4.8 Establishment and Treatment of the Acute Radiation‐Induced Lung Injury (RILI) Mouse Model

Mice were anesthetized with 1% sodium pentobarbital (50 mg/kg) and received a single 20 Gy X‐ray irradiation targeted to the right lung at a dose rate of 75 cGy/min using an X‐Rad 320 irradiator (Precision, USA). Treatments were started on the day of irradiation and given once daily for 4 weeks. Sham treatment was performed in all non‐irradiated and untreated groups.

### 4.9 Dose Justification

Dexamethasone (DXM) was administered via tail vein injection at 1 mg/kg once daily. The dose was selected based on clinical guidelines and previous studies on RILI [47, 59, 60]. The mouse dose was converted using FDA‐recommended body surface area normalization [61]. Animal dose = Human dose × (Human Km / Animal Km), where Km factors are 37 (human) and 3 (mouse). This dose corresponds to a human equivalent dose of ∼0.08 mg/kg/day, within the clinical range for dexamethasone (0.5–10 mg/day in adults, typically 0.007–0.14 mg/kg/day). HXF and NHXF were lyophilized and reconstituted in sterile water to 100 µg/mL (50 µg/mL for low‐dose group), then administered via once‐daily nebulized inhalation for 4 weeks. Under nose‐only exposure conditions (0.2 mL/min output rate, 1 L/min airflow, and 10 min exposure), the estimated pulmonary deposited dose was approximately 0.45 mg/kg per exposure.

### 4.10 Transcriptomic Sequencing

Total RNA was extracted using TRIzol reagent according to the manufacturer's protocol. RNA purity and concentration were assessed using a NanoDrop 2000 spectrophotometer (Thermo Scientific, USA), and RNA integrity was evaluated using an Agilent 2100 Bioanalyzer (Agilent Technologies, Santa Clara, CA, USA). Sequencing libraries were prepared with the VAHTS Universal V10 RNA‐seq Library Prep Kit (Premixed Version). Transcriptome sequencing was performed by Shanghai OE Biotech Co., Ltd. (Shanghai, China). Differentially expressed genes (DEGs) were identified using the thresholds |log_2_FC| > 1, FDR‐adjusted *q* < 0.05 (using the Benjamini–Hochberg procedure). Functional enrichment analysis, including KEGG, GO, and GSEA, was subsequently conducted.

### 4.11 Statistical Analysis

All statistical analyses and graphical representations were carried out using GraphPad Prism 9 software. The Shapiro‐Wilk test and Brown‐Forsythe test were used to assess normality and homogeneity of variance, respectively. Data meeting both assumptions (P ≥ 0.05) were analyzed using one‐way ANOVA followed by Tukey's multiple comparisons test. For datasets that failed to meet the assumptions of normality and/or homogeneity of variance, the Kruskal‐Wallis test followed by Dunn's multiple comparisons test was applied. Data are presented as mean ± SD or mean ± SEM as indicated in the figure legends. Statistical significance was defined as *p* < 0.05.

## Author Contributions

K.X., B.L., J.L. and M.C. conceived and designed the study. L.Q., J.C., X.C. and H.L. performed the experiments. Z.F., Y.M. and S.F. validated the experimental results. L.Q. analyzed and interpreted the data. L.Q., J.C. and X.C. prepared the figures. L.Q. and J.C. drafted the manuscript. P.Z. and M.C. revised and edited the manuscript. J.L. supervised the project. B.L., J.L. and M.C. provided resources and acquired funding. J.L. and M.C. administered the project.

## Funding

This work was supported by the Fundamental Research Funds for the Central Universities (ZYGX2021YGCX001); the Health Commission of Sichuan Province (ZH2023‐801,24QNMP038); the Scientific Research Project of Sichuan Medical Association (2024HR15) and the Transformation Foundation of Tianfu Jincheng Laboratory (2025ZH044).

## Conflicts of Interest

The authors declare no conflicts of interest.

## Supporting information




**Supporting File**: advs77472‐sup‐0001‐SuppMat.docx.

## Data Availability

The data that support the findings of this study are available from the corresponding author upon reasonable request.
