# Supplementary material for: A Huaxian Formula Nanoformulation Confers Protection Against Acute Radiation‐Induced Lung Injury by Targeting Ferroptosis
Source: Adv Sci (Weinh). 2026 Aug 29:e77472. Online ahead of print. doi: 10.1002/advs.77472 (PMC13525703; doi:10.1002/advs.77472)
Supplement: Supplementary file 1 [file ADVS-9999-e77472-s001.docx]

**Supporting Information**

Supporting Information is available from the Wiley Online Library or from the author.

An inhalable, nanoengineered herbal therapy achieves protection against acute radiation-induced lung injury via self-assembled nanomicelle–mediated pulmonary targeting and a previously unrecognized dual mechanism that attenuates oxidative stress while inhibiting ferroptosis in alveolar epithelial cells.

**A Huaxian Formula Nanoformulation Confers Protection against Acute Radiation-Induced Lung Injury by Targeting Ferroptosis**

*Li Quan, Junyang Chen, Xingmin Chen, Zengyi Fang, Yuanzhen Mi, Pingjin Zou, Shunlian Fu, Huakang Li, Ke Xu, Bing Lin *, Jinyi Lang* and Meihua Chen**

**Supporting Information**

**A Huaxian Formula Nanoformulation Confers Protection against Acute Radiation-Induced Lung Injury by Targeting Ferroptosis**

*Li Quan, Junyang Chen, Xingmin Chen, Zengyi Fang, Yuanzhen Mi, Pingjin Zou, Shunlian Fu, Huakang Li, Ke Xu, Bing Lin *, Jinyi Lang* and Meihua Chen**

**1 Materials and Methods**

*Encapsulation Efficiency (EE):* The encapsulation efficiency of NHXF-loaded micelles was determined by ultrafiltration coupled with LC-MS/MS analysis, using neoamygdalin, hesperidin, and baicalin as representative marker compounds. Two equal aliquots of NHXF micellar dispersion were prepared. For determination of total drug content (W_total), one aliquot was disrupted with a methanol/acetonitrile mixture (1:1, v/v), followed by vortexing and sonication for 5 min to ensure complete dissociation of micelles. After centrifugation, the supernatant was collected for LC-MS/MS analysis. For free drug content (W_free), the second aliquot was subjected to ultrafiltration using a 100 kDa centrifugal filter at 10,000 rpm for 15 min, and the resulting filtrate containing unencapsulated compounds was directly analyzed by LC–MS/MS. Encapsulation efficiency was calculated as: EE (%) = [(W_total − W_free) / W_total] × 100%.

*Nanoparticle Size and Zeta Potential Measurement:* The hydrodynamic diameter and zeta potential of nanoparticles were measured by dynamic light scattering and laser Doppler micro-electrophoresis, respectively, using a Zetasizer Nano ZS90 (Malvern, UK). HXF and NHXF samples were diluted with deionized water to an appropriate scattering intensity and loaded into polystyrene cuvettes or disposable folded capillary cells. All measurements were conducted at 37°C after a 2-min equilibration.

*Transmission Electron Microscopy (TEM) Observation:* The morphology of HXF and NHXF nanoparticles was characterized by TEM. Lyophilized samples were reconstituted in ultrapure water (1 mg/mL), and 10 µL aliquots were deposited onto 300-mesh carbon-coated copper grids for 10 min. The grids were rinsed with distilled water, negatively stained with 2% sodium phosphotungstate (10 s × 2), air-dried, and imaged using a Hitachi HT7800 TEM at 80 kV.

*Ultraviolet-Visible (UV-Vis) Absorption Spectroscopy:* UV–Vis absorption spectra of PEG–PCL, HXF, NHXF, neoamygdalin, hesperidin, and baicalin were recorded over 200–800 nm. PEG–PCL, HXF, and NHXF were dissolved in ultrapure water, whereas Neoamygdalin, Hesperidin, and Baicalin were dissolved in DMSO. The corresponding solvents were used as blanks, and the spectra were analyzed using GraphPad Prism software.

*Identification of NHXF Micelle Components via HPLC-MS:* To analyze NHXF composition, 150 µL of micellar dispersion was mixed with an equal volume of aqueous internal standards (2 µg/mL), vortexed, sonicated in an ice-water bath for 60 min, and centrifuged (12,000 rpm, 10 min, 4°C). The supernatant (120 µL) was filtered and injected for UPLC-MS analysis. Separation was performed on an ACQUITY UPLC HSS T3 column (100 × 2.1 mm, 1.8 µm) at 45°C using 0.1% formic acid in water and acetonitrile (0.35 mL/min, 2 µL injection). Data were processed with XCMS for peak detection, integration, and retention time alignment, and compounds were tentatively identified by accurate mass and MS/MS fragment matching against the LuMet-TCM database.

*In Vitro Release Study:* NHXF micellar dispersion (2 mL) was loaded into a dialysis bag (MWCO 14 kDa) and immersed in 50 mL of simulated alveolar fluid (SAF, pH 7.4, containing 0.05% DPPC) at 37 °C with shaking at 100 rpm. At predetermined time points (0.5, 1, 2, 3, 6, 12, 18, and 24 h), 1 mL of release medium was collected and replaced with an equal volume of fresh prewarmed SAF. The concentrations of Neoamygdalin, Hesperidin, and Baicalin in the release medium were quantified by LC–MS/MS, and the cumulative release percentage was calculated after volume correction.

*Aerodynamic Particle Size Distribution (APSD):* The APSD of NHXF was characterized using a 10-stage Mini MOUDI™ impactor (Model 135-10B, TSI Inc., USA) operated at a flow rate of 2 L/min. Following nebulization, aerosol deposits collected on each stage and the terminal filter were quantified, and the corresponding mass percentage and cumulative mass percentage distributions were plotted according to the aerodynamic cut-off diameters (0.056–10 μm). The mass median aerodynamic diameter (MMAD) and geometric standard deviation (GSD) were calculated from the cumulative distribution curve.

*Cell Viability Assay:* RLE-6TN cells were seeded in 96-well plates at 5 × 10³ cells/well and treated with HXF or NHXF (0–100 μg/mL) for 24 h prior to 10 Gy X-ray irradiation (200 cGy/min). LLC and A549 cells were seeded at 3 × 10³ cells/well and pretreated with NHXF (100 μg/mL) for 24 h before irradiation under the same conditions. Cell viability was assessed using a CCK-8 assay, and absorbance was measured at 450 nm. Viability was calculated as [(OD_treatment − OD_blank)/ (OD_control − OD_blank)] × 100%.

*Colony Formation Assay:* RLE-6TN, LLC, and A549 cells were seeded into 6-well plates at densities of 3 × 10², 1 × 10³, and 6 × 10² cells per well, respectively. RLE-6TN cells were assigned to Ctrl, NHXF, IR, IR + PEG–PCL, IR + HXF, IR + NHXF-L, and IR + NHXF-H groups, whereas LLC and A549 cells were assigned to Ctrl, NHXF, IR, and IR + NHXF groups. After 24 h pretreatment with the indicated formulations, cells were exposed to 10 Gy X-ray irradiation and cultured for an additional 10 days with medium renewal every 3 days. Colonies were then fixed, stained with crystal violet, imaged, and quantified using ImageJ software.

*Wound Healing (Scratch) Assay:* Cells were seeded in 6-well plates at 3 × 10⁵ cells per well and allowed to adhere for 24 h. A uniform scratch was generated using a sterile 200 µL pipette tip, followed by 10 Gy X-ray irradiatio. Wound closure was imaged at 0, 24, and 48 h using an inverted microscope, and the remaining wound areas were quantified using ImageJ to assess cell migration.

*RT-qPCR Analysis:* Total RNA was extracted from RLE-6TN cells and lung tissues using TRIzol reagent (Invitrogen, USA) according to the manufacturer’s instructions, followed by reverse transcription into cDNA using a reverse transcription kit. Quantitative PCR was performed using SYBR Green Master Mix on a real-time PCR system. Gene expression levels were normalized to β-actin and calculated using the 2^⁻ΔΔCt^ method. Details of the primer sequences used in this study are provided in Table S3 in the Supporting Information.

*Hematoxylin and Eosin (H&E) Staining:* Tissue samples were fixed in 4% paraformaldehyde for 24 h, dehydrated through graded ethanol, cleared in xylene, and embedded in paraffin. Sections (3 µm) were cut, deparaffinized, rehydrated, and stained with hematoxylin and eosin. After dehydration and mounting with neutral resin, morphological changes were examined under a bright-field microscope.

*Immunohistochemistry (IHC):* Following deparaffinization and rehydration, tissue sections were subjected to antigen retrieval in Tris–EDTA buffer (pH 8.0). Endogenous peroxidase activity was quenched with 3% H₂O₂, and non-specific binding was blocked with goat serum. Sections were incubated overnight at 4 °C with primary antibodies against MPO (1:1000; Abcam, USA), 8-OHdG (1:300; Bioss, China), and Ki67 (1:500; Abcam, USA). After washing, sections were incubated with HRP-conjugated secondary antibodies (1:5000; goat anti-rabbit/mouse IgG) for 2 h at room temperature. Signals were visualized using DAB and counterstained with hematoxylin. Slides were dehydrated, cleared, mounted, and scanned using a VENTANA DP 200 system (Roche, Switzerland). Positive staining areas were quantified using ImageJ software.

*Immunofluorescence Staining:* Sections were deparaffinized, rehydrated, and subjected to antigen retrieval in Tris-EDTA buffer (pH 8.0), followed by blocking with goat serum. Sections were incubated overnight at 4°C with anti-MPO primary antibody (1:2000; Abcam, USA) and then with fluorophore-conjugated secondary antibody (1:5000; Abcam, USA) for 1 h at room temperature in the dark. Nuclei were counterstained with DAPI, and slides were mounted with antifade medium. Fluorescence images were acquired using an Olympus SLIDEVIEW VS200 microscope and quantified with ImageJ.

*Flow Cytometry Analysis:* Peripheral blood and lung neutrophils were quantified by flow cytometry. Blood was collected via the retro-orbital sinus, and lungs were harvested, mechanically dissociated, and digested with collagenase IV to generate single-cell suspensions, which were filtered through a 70 µm strainer and treated with red blood cell lysis buffer. Cells were stained with fluorescent antibodies against CD11b and Ly-6G (Thermo Fisher, USA) at 4°C for 30 min in the dark. Data were acquired on a Cytek Aurora/Northern Lights spectral flow cytometer, and neutrophils were gated as CD11b⁺Ly-6G⁺ cells.

*Enzyme-Linked Immunosorbent Assay (ELISA):* Levels of IL-6, IL-1β, and TNF-α in serum (1:5 dilution) and bronchoalveolar lavage fluid (BALF) supernatants, as well as TGF-β1, MCP-1, IL-10, and IFN-γ in serum, were quantified using commercial ELISA kits (LabLead, China) according to the manufacturer's instructions.

*In Vivo Safety Evaluation:* After 4 weeks of nebulization, all mice were euthanized, and major organs (heart, liver, spleen, kidneys) were collected for H&E histological analysis. Serum biochemical parameters, including ALT, AST, BUN, CREA, CK, CK-MB, LDH, and α-HBDH, as well as hematological parameters (RBC, WBC, PLT, HGB, and NEUT), were analyzed to evaluate the systemic safety.

*Establishment and Treatment of a Subcutaneous LLC Tumor Model:* Male C57BL/6J mice were subcutaneously inoculated with 5 × 10⁴ LLC cells into the shaved posterior aspect of the right hind limb. When tumors reached approximately 50 mm³ (10–14 days post-inoculation), mice were randomly assigned to four groups: Ctrl, NHXF, IR, and IR + NHXF. Mice in the IR and IR + NHXF groups received localized tumor irradiation on days 0, 2, and 4 at a dose of 7 Gy per fraction. Treatments were initiated on day 0 and continued throughout the study. Ctrl and IR mice received saline via nebulization and peritumoral injection, whereas NHXF and IR + NHXF mice received NHXF via nebulization and peritumoral injection at a dose of 0.45 mg/kg.

*Western Blot Analysis:* Western blotting was performed following standard procedures. Cell lysates were separated by SDS-PAGE and transferred onto 0.45 μm PVDF membranes. After blocking (Oriscience, China), membranes were incubated overnight at 4°C with primary antibodies: FTH1, ACSL4 (1:1000, ABclonal, China), FTL1, GPX4 (1:1000, Zenbio, China), and β-actin (1:20,000, ABclonal, China), followed by HRP-conjugated secondary antibody (Zenbio, China) for 1 hour at room temperature. Protein bands were detected using chemiluminescence.

*Detection of Intracellular Fe²⁺ using FerroOrange Probe:* Cells were seeded at 2×10⁵ per well in 48-well plates and pre-treated for 24 hours before a single 10 Gy X-ray irradiation. At 24 hours post-irradiation, cells were stained with FerroOrange (Dojindo, Japan) to detect intracellular Fe²⁺, and fluorescence was imaged by confocal microscopy and quantified using ImageJ.

*Measurement of Malondialdehyde (MDA):* Cells (1×10⁵/well) in 6-well plates were pre-treated with NHXF for 24 hours before 10 Gy X-ray irradiation. At 24 hours post-irradiation, supernatants were collected following the manufacturer’s protocol (Dojindo, Japan), and MDA levels were measured via fluorescence (Ex 540 nm/Em 590 nm) using a BioTek Cytation 5 microplate reader and quantified against a standard curve.

**Supplementary Figures**

**Figure S1.** The Tyndall effect phenomenon demonstrates the formation of NHXF nanoaggregates across a concentration gradient, with water serving as the negative control.

**Figure S2.** The BPC of the water extract of NHXF granules produced by UHPLC-MS. The x-axis represents retention time, and the y-axis represents chromatographic peak

abundance. (A) Positive ion base peak mass spectrum; (B) Negative ion base peak mass spectrum.

**Figure S3.** UV-Vis absorption spectra of neoamygdalin, hesperidin, baicalin, and NHXF under simulated physiological conditions (37 °C, pH 7.4) in the wavelength range of 200–800 nm.

**Figure S4.** Cumulative mass distribution percentage of NHXF under different aerodynamic diameter cut conditions.

**Figure S5.** Physicochemical characterization of NHXF nanomicelles before and after nebulization, including particle size (nm), polydispersity index (PDI), and zeta potential (mV). Data are presented as mean ± SD (n = 3). Statistical analysis was performed using t tests.; ns, not significant.

**Figure S6. Evaluation of NHXF and HXF Cytotoxicity in RLE-6TN Cells.** RLE-6TN cells were treated with (A) NHXF or (B) HXF over a concentration range of 0 to 100 µg/mL for 24, 48, and 72 hours. Cell viability was quantified using the CCK-8 assay. Data are expressed as mean ± SEM (n = 3 independent experiments). Statistical analysis was performed using one-way ANOVA with Tukey’s multiple comparisons test. *P < 0.05, **P < 0.01, ****P < 0.0001; ns, not significant.

**Figure S7. RT-qPCR assessment of radiation-induced DNA damage response and double-strand break repair-associated genes.** Relative mRNA expression levels of (A) *Atm*, (B) *Tp53bp1*, (C) *H2ax*, and (D) *Cdkn1a* as determined by RT-qPCR. Data are presented as mean ± SEM (n = 3 independent experiments). Statistical analysis was performed using one-way ANOVA with Tukey’s multiple comparisons test. *P < 0.05, **P < 0.01, ***P < 0.001, ****P < 0.0001; ns, not significant.

**Figure S8.** **Evaluation of cell migration under different treatments via a wound healing assay.** (A) Representative images of the wound area at 0, 24, and 48 hours post-scratching, illustrating the migration over time. Quantitative analysis of the relative wound closure area at (B) 24 hours and (C) 48 hours. Scale bars, 200 μm. Data are presented as mean ± SEM (n = 3 independent experiments). Statistical analysis was performed using one-way ANOVA with Tukey’s multiple comparisons test. *P < 0.05, **P < 0.01, ***P < 0.001, ****P < 0.0001; ns, not significant.

**Figure S9.** The temporal trend of body weight changes in mice following irradiation exposure. Data are presented as mean ± SD (n = 5 per group). Statistical analysis was performed using one-way ANOVA with Tukey’s multiple comparisons test.

**Figure S10.** Representative image of a Diff-Quik stained cytospin preparation of mouse bronchoalveolar lavage fluid (BALF) and quantification of total cell counts in BALF. Scale bar, 50 µm. Data are presented as mean ± SD (n = 5 per group). Statistical analysis was performed using one-way ANOVA with Tukey’s multiple comparisons test. **P < 0.01, ****P < 0.0001; ns, not significant.

**Figure S11. RT-qPCR analysis of inflammatory chemokines in RLE-6TN cells and lung tissues.** Relative mRNA expression levels of *Cxcl1*, *Cxcl2*, and *Cxcl5* in RLE-6TN cells and lung tissues as determined by RT-qPCR. Data are presented as mean ± SEM for RLE-6TN cell experiments (n = 3 independent experiments) and mean ± SD for lung tissue experiments (n = 5 per group). Statistical analysis was performed using one-way ANOVA with Tukey’s multiple comparisons test. *P < 0.05, **P < 0.01, ***P < 0.001, ****P < 0.0001; ns, not significant.

**Figure S12.** Representative flow cytometry plots showing the gating strategy for neutrophils (identified as CD11b⁺Ly6G⁺ cells) in mouse blood and quantitative analysis of the CD11b⁺Ly6G⁺ neutrophil population. Data are presented as mean ± SD (n = 3 technical replicates derived from pooled samples of 5 mice). Statistical analysis was performed using one-way ANOVA with Tukey’s multiple comparisons test. **P < 0.01, ***P < 0.001, ****P < 0.0001; ns, not significant.

**Figure S13. ELISA quantification of profibrotic, inflammatory, and immunoregulatory factors in serum.** Concentrations of (A) TGF-β1, (B) MCP-1, (C) IFN-γ, and (D) IL-10 in serum were measured by ELISA. Data are presented as mean ± SD (n = 5 per group). Statistical significance was determined using one-way ANOVA followed by Tukey’s multiple-comparisons test. *P < 0.05, ***P < 0.001, ****P < 0.0001; ns, not significant.

**Figure S14.** ELISA quantification of pro-inflammatory cytokines TNF-α, IL-6, and IL-1β in bronchoalveolar lavage fluid (BALF). Data are presented as mean ± SD (n = 3 technical replicates derived from pooled samples of 5 mice). Statistical analysis was performed using one-way ANOVA with Tukey’s multiple comparisons test. **P < 0.01, ***P < 0.001, ****P < 0.0001; ns, not significant.

**Figure S15. Hematological analysis.** The evaluated parameters included (A) red blood cell count (RBC), (B) white blood cell count (WBC), (C) platelet count (PLT), (D) hemoglobin (HGB), and (E) neutrophil percentage (NEUT%). Data are presented as mean ± SD (n = 5 per group). Statistical significance was determined by one-way ANOVA followed by Tukey’s multiple-comparisons test. *P < 0.05, **P < 0.01, ***P < 0.001, ****P < 0.0001; ns, not significant.

**Figure S16. Serum biochemical analysis.** Levels of (A) aspartate aminotransferase (AST), (B) alanine aminotransferase (ALT), (C) urea, (D) creatinine (Cr), (E) creatine kinase (CK), (F) creatine kinase isoenzyme MB (CK-MB), (G) lactate dehydrogenase (LDH), and (H) alpha-hydroxybutyrate dehydrogenase (α-HBDH). Data are presented as mean ± SD (n = 5 per group). Statistical analysis was performed using one-way ANOVA with Tukey’s multiple comparisons test; ns, not significant.

**Figure S17. In vivo safety assessment.** Representative hematoxylin and eosin (H&E) stained sections of major organs (heart, liver, spleen, and kidney) from mice. Scale bars, 100 μm.

**Figure S18. In vitro anti-tumor effect of NHXF combined with radiotherapy.** (A) Cell viability of LLC cells and (B) A549 cells at 0, 12, 24, 36, 48, 60, and 72 h post-irradiation, as determined by the CCK-8 assay. (C) Representative images of colony formation assays in LLC cells and (E) in A549 cells; (D, F) Corresponding quantitative analysis of colony numbers. Data in (A-B) and (D-F) are presented as mean ± SEM (n = 3 independent experiments). Statistical analysis was performed using one-way ANOVA followed by Tukey's multiple comparison test; ns, not significant.

**Figure S19. In vivo anti-tumor effect of NHXF combined with radiotherapy.** (A) Schematic diagram of the in vivo experimental design. (B) Representative images of ex vivo tumors. Scale bars = 1 cm. (C) Tumor growth curves. (D) Representative images of H&E staining of tumor tissues. Scale bars = 50 μm. (E) Statistical analysis of tumor mass. (F) Representative images of Ki67 immunohistochemical staining of tumor tissues and (G) quantitative analysis results. Scale bars, 50 μm. Data in (C), (E), and (G) are presented as mean ± SD (n = 6 per group). Statistical analysis was performed using one-way ANOVA followed by Tukey's multiple comparison test; ns, not significant.

**Figure S20.** Volcano plot illustrating the differentially expressed genes in IR versus control (DEGs; |log₂FC| > 1, FDR-adjusted q < 0.05).

**Figure S21.** Protein–protein interaction (PPI) network demonstrating the regulatory effects of NHXF on proteins involved in the ferroptosis pathway. Proteins highlighted in red represent up-regulated expressions, while those in blue indicate down-regulation.

**Figure S22.** (A) Representative images of intracellular reactive oxygen species (ROS) levels detected using the DCFH-DA probe (green) following treatment with the ferroptosis inhibitor Fer-1, and (B) following treatment with the ferroptosis agonist IKE. Scale bars, 50 μm. (C, D) Corresponding quantitative analysis of fluorescence intensity. Data in (C) and (D) are presented as mean ± SEM (n = 3 independent experiments). Statistical analysis was performed using one-way ANOVA followed by Tukey's multiple comparison test. **P < 0.01, ****P < 0.0001; ns, not significant.

**Supplementary Tables**

**Table S1** The major compound constituents of NHXF

| Peak | Formula | Metabolites | Ion  mode | Retention time (min) |
| --- | --- | --- | --- | --- |
| 1 | C_12_H_22_O_11_ | Turanose | NEG | 0.897 |
| 2 | C_21_H_18_O_11_ | Baicalin | NEG | 20.504 |
| 3 | C_5_H_9_NO_2_ | (R)-pyrrolidine-2-carboxylic acid | POS | 0.786 |
| 4 | C_22_H_20_O_11_ | Wogonoside | POS | 21.073 |
| 5 | C_7_H_13_NO_2_ | Stachydrine | POS | 0.865 |
| 6 | C_28_H_34_O_15_ | Hesperidin | NEG | 19.85 |
| 7 | C_20_H_27_NO_11_ | Neoamygdalin | NEG | 12.058 |
| 8 | C_21_H_18_O_11_ | Glychionide A | NEG | 20.844 |
| 9 | C_7_H_7_NO_2_ | Trigonelline | POS | 0.786 |
| 10 | C_15_H_10_O_5_ | Sulfuretin | POS | 20.507 |
| 11 | C_27_H_45_NO_3_ | Peimine | POS | 19.785 |
| 12 | C_27_H_32_O_16_ | Hydroxysafflor yellow A | NEG | 11.911 |
| 13 | C_26_H_28_O_13_ | Chrysin 6-C-arabinoside 8-C-glucoside | NEG | 17.406 |
| 14 | C_22_H_20_O_12_ | 5,8-Dihydroxy-6,-methoxyflavone-7-O-glucuronide | POS | 20.957 |
| 15 | C_21_H_22_O_9_ | Liquiritin | NEG | 16.877 |
| 16 | C_6_H_11_NO_2_ | Hygric acid | POS | 1.002 |
| 17 | C_9_H_10_O_5_ | Danshensu | NEG | 7.043 |
| 18 | C_24_H_42_O_21_ | Maltotetraose | POS | 1.089 |
| 19 | C_27_H_32_O_14_ | Narirutin | NEG | 18.678 |
| 20 | C_10_H_13_N_5_O_5_ | Crotonoside | POS | 5.749 |
| 21 | C_19_H_30_O_8_ | Roseoside | NEG | 13.684 |
| 22 | C_42_H_62_O_16_ | glycyrrhizinate | POS | 24.629 |
| 23 | C_27_H_45_NO_3_ | Isoverticine | POS | 20.706 |
| 24 | C_42_H_62_O_17_ | Licorice saponin G2 | NEG | 23.348 |

**Table S2.** The herbs composition of HXF.

| Herb name | Plant name | Dose |
| --- | --- | --- |
| Huangqi | *Astragalus mongholicus* | 30 g |
| Zhuru | *Bambusa tuldoides* | 20 g |
| Baihe | *Lilium lancifolium* | 20 g |
| Maidong | *Ophiopogon japonicus* | 20 g |
| Sangbaipi | *Morus alba* | 20 g |
| Jinyinhua | *Lonicera japonica* | 20 g |
| Pipaye | *Eriobotrya japonica* | 20 g |
| Fuling | *Poria cocos* | 20 g |
| Danshen | *Salvia miltiorrhiza* | 20 g |
| Huangqin | *Scutellaria baicalensis* | 15 g |
| Fabanxia | *Pinellia ternata* | 10 g |
| Chenpi | *Citrus reticulata* | 10 g |
| Shenggancao | *Glycyrrhiza uralensis* | 10 g |
| Honghua | *Carthamus tinctorius* | 5 g |
| Sanqi | *Panax notoginseng* | 5 g |

*All herbs were obtained from the Affiliated Hospital of Chengdu University of Traditional Chinese Medicine and provided by Sichuan New Green Pharmaceutical Technology Development Co., Ltd.

**Table S3** The primer of RT-qPCR.

| Genes | Forward Primer (5'to3') | Reverse Primer (5'to3') |
| --- | --- | --- |
| *Atm* (rat) | TCACCTTAAGGGTTCTCGTCG | GAACGTGCATCCTCACCTCA |
| *H2ax* (rat) | TCCCAGGCCTCTCAGGAGTA | AAGGGTAAAGACGCGAGGAC |
| *Tp53bp1* (rat) | CCACACCTGTCAGGACGAAC | GCAGGTCTGCTTCCATCACT |
| *Cdkn1a* (rat) | TCCTTGGAATTTCAGGCCACAG | AGACGACGGCATACTTTGCT |
| *Cxcl1* (rat) | ACTCAAGAATGGTCGCGAGG | CTTGGGGACACCCTTTAGCA |
| *Cxcl2* (rat) | CATCAGGGTACAGGGGTTGTT | GTCACCGTCAAGCTCTGGAT |
| *Cxcl5* (rat) | CATATCCCGAACCGGTGCTT | CCGAGAAAGGAGCAGCTTGAG |
| *β-actin* (rat) | CCGCGAGTACAACCTTCTTG | CGTCATCCATGGCGAACTGG |
| *Cxcl1* (mouse) | CTGGGATTCACCTCAAGAACATC | CAGGGTCAAGGCAAGCCTC |
| *Cxcl2* (mouse) | CCAACCACCAGGCTACAGG | GCGTCACACTCAAGCTCTG |
| *Cxcl5* (mouse) | TCCAGCTCGCCATTCATGC | TTGCGGCTATGACTGAGGAAG |
| *β-actin* (mouse) | CACTGTCGAGTCGCGTCC | TCATCCATGGCGAACTGGTG |
